# Generalizability of A Neural Network Model for Circadian Phase Prediction in Real-World Conditions

**DOI:** 10.1038/s41598-019-47311-4

**Published:** 2019-07-29

**Authors:** Julia E. Stone, Andrew J. K. Phillips, Suzanne Ftouni, Michelle Magee, Mark Howard, Steven W. Lockley, Tracey L. Sletten, Clare Anderson, Shantha M. W. Rajaratnam, Svetlana Postnova

**Affiliations:** 1 Cooperative Research Centre for Alertness, Safety and Productivity, Melbourne, Victoria Australia; 2 School of Psychological Sciences and Turner Institute for Brain and Mental Health, Faculty of Medicine, Nursing & Health Sciences, Monash University, Clayton, Victoria Australia; 3 Division of Sleep and Circadian Disorders, Departments of Medicine and Neurology, Brigham and Women’s Hospital, Boston, Massachusetts USA; 4 Division of Sleep Medicine, Harvard Medical School, Boston, Massachusetts USA; 5 Institute for Breathing and Sleep, Austin Health, Victoria, Australia; 6 School of Physics, University of Sydney, Sydney, New South Wales Australia

**Keywords:** Machine learning, Circadian rhythms and sleep

## Abstract

A neural network model was previously developed to predict melatonin rhythms accurately from blue light and skin temperature recordings in individuals on a fixed sleep schedule. This study aimed to test the generalizability of the model to other sleep schedules, including rotating shift work. Ambulatory wrist blue light irradiance and skin temperature data were collected in 16 healthy individuals on fixed and habitual sleep schedules, and 28 rotating shift workers. Artificial neural network models were trained to predict the circadian rhythm of (i) salivary melatonin on a fixed sleep schedule; (ii) urinary aMT6s on both fixed and habitual sleep schedules, including shift workers on a diurnal schedule; and (iii) urinary aMT6s in rotating shift workers on a night shift schedule. To determine predicted circadian phase, center of gravity of the fitted bimodal skewed baseline cosine curve was used for melatonin, and acrophase of the cosine curve for aMT6s. On a fixed sleep schedule, the model predicted melatonin phase to within ± 1 hour in 67% and ± 1.5 hours in 100% of participants, with mean absolute error of 41 ± 32 minutes. On diurnal schedules, including shift workers, the model predicted aMT6s acrophase to within ± 1 hour in 66% and ± 2 hours in 87% of participants, with mean absolute error of 63 ± 67 minutes. On night shift schedules, the model predicted aMT6s acrophase to within ± 1 hour in 42% and ± 2 hours in 53% of participants, with mean absolute error of 143 ± 155 minutes. Prediction accuracy was similar when using either 1 (wrist) or 11 skin temperature sensor inputs. These findings demonstrate that the model can predict circadian timing to within ± 2 hours for the vast majority of individuals on diurnal schedules, using blue light and a single temperature sensor. However, this approach did not generalize to night shift conditions.

## Introduction

Circadian rhythms of alertness, cognitive performance, and sleepiness are regulated by a master circadian pacemaker in the suprachiasmatic nuclei^1–3^. This circadian pacemaker generates a near-24 h endogenous rhythm, which is entrained to the 24-hour day by light^4,5^. Measuring or predicting circadian timing is valuable for predicting when workers may be at increased risk of performance failures, as well as for pinpointing the correct timing of circadian interventions such as light therapy for circadian rhythm sleep disorders^6–9^.

The current gold standard method for assessing the phase of the central circadian pacemaker is the melatonin rhythm, measurable from plasma or saliva, or its urinary metabolite, 6-sulphatoxymelatonin (aMT6s)^10,11^. However, measuring melatonin in the field is costly and logistically challenging due to requiring samples at multiple time-points. Moreover, it typically requires days to weeks to assay samples. Consequently, current methods for assessing circadian phase are largely unsuitable for real-time monitoring. Practical, non-invasive methods for assessing circadian phase outside of the laboratory are needed.

Recently, modeling approaches have been developed to predict circadian phase from ambulatory measures under normal living conditions, with a mean absolute error ranging from 19 to ~87 minutes^12–17^. To date, the most accurate of these published methods used artificial neural network modeling^15^, which is a form of supervised machine learning that has been used for pattern recognition in physiological data^18^, including in the fields of sleep, alertness, and fatigue^19,20^. The neural network approach is powerful because the model is capable of learning complex non-linear relationships between a series of input-output pairs^21^.

Kolodyazhniy, *et al*.^15^ developed a neural network model that takes light exposure and skin temperature data as inputs to predict salivary melatonin phase. Healthy young male participants (*n* = 25) wore ambulatory recording equipment over seven days while maintaining a fixed sleep schedule, prior to circadian phase assessment under laboratory constant routine conditions. Using this method, peripheral skin temperature measured over distal and proximal skin sites and blue light irradiance measured at eye level provided the most precise estimates of melatonin phase (mean absolute error of 19 ± 13 minutes) compared to a multivariate non-linear regression approach using the same inputs, or univariate approaches such as phase estimates based on ambulatory core body temperature or sleep timing^14,15^.

The neural network model has, however, only been tested in healthy male participants living on a fixed diurnal sleep schedule. It is unclear whether this approach is able to predict circadian phase when individuals are on other diurnal sleep schedules, or when the primary light-dark (or sleep-wake) signal is uncoupled from the circadian system, as is observed in rotating shift workers prior to adaptation to the new work schedule^22–26^. In shift workers in the field it is impractical to measure melatonin in plasma or saliva, as the assessment method requires strict control of sleep and light exposure. Thus, existing models need to be extended to predict the acrophase of urinary aMT6s, which is the current gold standard measure of circadian phase in the field. Further testing is therefore required to validate and extend the neural network method for use in a wider range of conditions, including circadian misalignment.

The current study aimed to determine the generalizability of the artificial neural network model under a range of real-world sleep-wake conditions. In particular, we aimed to (i) replicate prior findings to predict salivary melatonin phase on a fixed sleep-wake schedule; (ii) extend the method to predict urinary aMT6s acrophase for individuals with diurnal sleep-wake patterns, including real-world shift workers on a day-active schedule; and (iii) extend the method to predict urinary aMT6s acrophase for rotating shift workers.

## Results

**Figure 1:**
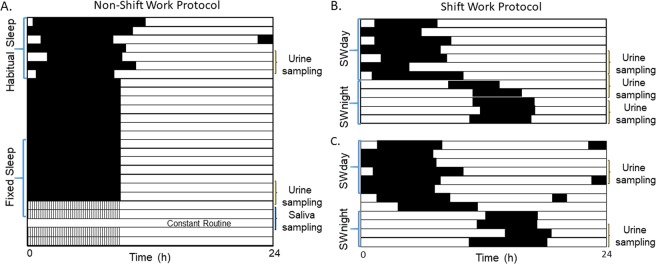
Example raster plots for the protocols used. (**A**) Raster plot of sleep timing for a participant on the non-shift work protocol; black bars indicate sleep in the at-home environment, black indicates sleep in the laboratory. Approximately one week of habitual sleep, and the second of two weeks of fixed sleep were used as inputs into the model. (**B**,**C**) Raster plots for two participants on the shift work protocol, working a day-active schedule followed by a series of night shifts. SWday indicates the day/evening shift schedule; SWnight indicates the night shift schedule. Number of day/evening shifts or days off varied between participants. Number of night shifts ranged from 3–7.

Data for this study came from two protocols illustrated in Fig. 1. First, a non-shift work study where healthy individuals were monitored over a week of habitual sleep (HS dataset), followed by two weeks of fixed sleep (FS dataset) with an 8:16 h sleep:wake schedule. At the end of the 3 weeks, participants came to the laboratory, where salivary melatonin was assessed under constant routine conditions for 40 h. In addition, urinary aMT6s was collected at the end of the HS and FS episodes, for 48 h each time. Second, a shift work study where rotating shift workers were monitored over a series of day/evening shifts and days off (SWday dataset) followed by three or more consecutive night shifts (SWnight dataset). During both studies, red, green, and blue light irradiances were measured continuously using wrist-worn actigraph devices, and skin temperature was monitored using wireless temperature sensors worn on the non-dominant wrist (shift work study), and over 11 skin sites including shoulders, sternum, wrist, thighs, ankles, and feet (non-shift work study). See Methods for more information.

### Reference circadian phase across datasets

Sleep and circadian timing for each dataset are summarized in Table 1. After the FS protocol, salivary melatonin and urinary aMT6s phase times were strongly correlated (*r*^2^ = 0.77, *p* = 0.05), with urinary aMT6s acrophase occurring on average 1.13 (±0.63 SD) hours later than melatonin phase measured from saliva. aMT6s acrophase times on the HS and FS schedules were also correlated (*n* = 12, *r*^2^ = 0.45, *p* = 0.05), with acrophase on the HS schedule occurring on average 0.54 hours later than on the FS schedule.

**Table 1:** Dataset characteristics by sleep schedule

	Fixed Sleep						Habitual Sleep					Shift Work Diurnal					Shift Work Nights			
	n	Mean	SD	Min	Max	n	Mean	SD	Min	Max	N	Mean	SD	Min	Max	n	Mean	SD	Min	Max
Sex (M, F)	12, 3					11, 3					7, 22					4, 15				
Age (years)	15	26.01	6.38	20.70	45.54	14	26.35	6.62	20.70	45.54	29	31.83	7.69	23.00	58.00	19	34.89	10.86	24.00	64.00
BMI (kg/m^2^)	15	22.70	2.52	19.20	28.22	14	22.55	2.61	19.20	28.22	29	24.23	4.14	15.90	32.00	19	24.38	3.60	15.90	29.70
MEQ Score	15	39.07	3.24	34.00	45.00	14	38.57	3.59	34.00	45.00	29	38.07	6.50	23.00	52.00	19	39.11	6.57	28.00	52.00
Mean Sleep Onset (h)	15	23.30	0.72	22.25	24.29	14	24.06	0.88	22.72	25.42	29	23.44	0.48	22.63	24.44	19	9.56	1.65	7.19	14.47
Mean Sleep Offset (h)	15	6.86	0.71	5.78	7.92	14	7.68	1.09	5.71	9.45	29	7.15	1.20	5.60	10.09	19	15.05	1.81	11.18	17.66
Mean Mid-sleep Time (h)	15	3.08	0.71	2.01	4.07	14	3.87	0.84	2.22	5.18	29	3.30	0.75	2.34	5.27	19	12.30	1.37	9.19	15.09
Salivary Melatonin Phase (h)	15	2.57	0.86	0.42	4.02	—					—					—				
Urinary aMT6s Acrophase (h)	13	3.72	1.21	0.92	5.68	14	4.24	1.05	2.40	6.37	29	3.90	1.77	20.22	6.75	19	5.16	2.85	22.95	14.18
Bedtime Phase Angle (h)	13	−4.43	1.26	−6.43	−2.61	14	−4.18	1.35	−6.26	−1.60	29	−4.33	2.04	−7.12	4.22	19	4.14	3.44	−5.66	9.95
Waketime Phase Angle (h)	13	3.12	1.30	1.21	5.28	14	3.43	1.08	1.68	5.43	29	3.38	2.38	−1.14	13.87	19	9.64	3.10	1.22	14.23
Length of pre-processed data set (days)	13	5.84	0.63	4.65	7.60	14	5.40	1.04	3.02	6.12	29	4.55	1.79	1.56	8.90	19	3.36	0.86	1.67	5.29

Note: Phase angle calculated using aMT6s acrophase. N = 2 on FS do not have aMT6s acrophase (only melatonin), and thus were excluded from this calculation. BMI = body mass index, MEQ = Horne & Ostberg Composite Morningness-Eveningness Questionnaire, aMT6s = urinary 6-sulphatoxymelatonin.

There was greater variability in melatonin timing on the shift work protocol, with a wider range in aMT6s acrophase on both the diurnal and night shift schedules (Table 1). There was no significant difference between mean aMT6s acrophase time on the diurnal (4.60 ± 3.26 h) or night shift schedules (6.68 ± 4.78 h; *f*(46) = 1.97, *p* = 0.17). However, aMT6s acrophase time was significantly later on the night shift schedule (6.68 ± 4.78 h) compared to the non-shift work protocol (combined HS + FS: 3.98 ± 1.22 h; *f*(44) = 8.10, *p* = 0.007).

### Artificial neural network model predictions

**Figure 2:**
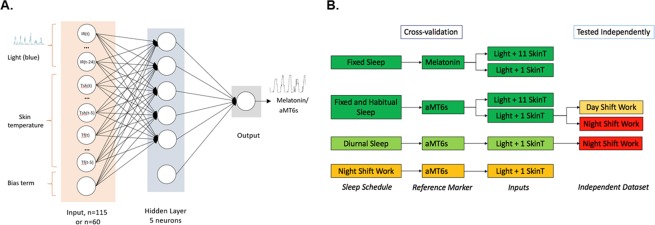
(**A**) Schematic of neural network structure reproduced from^15^. Blue light irradiance and skin temperature data were inputs to a two-layer perceptron with a hidden layer of 5 neurons, plus a bias term. The input variables included a light variable (blue irradiance) with lags of 0, 1, 2, …, 24 h with either six skin temperature variables (shoulders, sternum, wrists, thighs, ankles, feet) or one skin temperature variable (wrist) with lags of 0, 1, 2, …, 5 h, plus a bias term. Networks using light plus 6 skin temperature variables had 115 inputs, plus a bias term, resulting in a total of 586 adjustable weights. Networks using light and 1 skin temperature variable had 60 inputs, plus a bias term, resulting in a total of 311 adjustable weights. Output was either predicted melatonin concentration or aMT6s excretion rate. (**B**) Schematic of models trained using cross-validation by sleep schedule: fixed sleep trained on melatonin profiles using light with 1 or 11 skin temperature sensors; fixed and habitual sleep trained on aMT6s profiles using light and 1 or 11 skin temperature sensors; diurnal sleep (fixed, habitual, and shift work day schedule datasets) trained on aMT6s profiles using light and 1 skin temperature sensor; night shift schedule trained on aMT6s using light and 1 skin temperature sensor. Additionally, the fixed and habitual sleep network using light and 1 skin temperature sensor was independently tested on shift work datasets (day and night shift schedules); and the diurnal sleep model was independently tested on the night shift work datasets. Colors indicate model performance based on mean absolute error: dark green < 55 minutes; light green < 65 minutes; light orange < 85 minutes; dark orange < 145 minutes; red > 145 and < 440 minutes.

**Figure 3:**
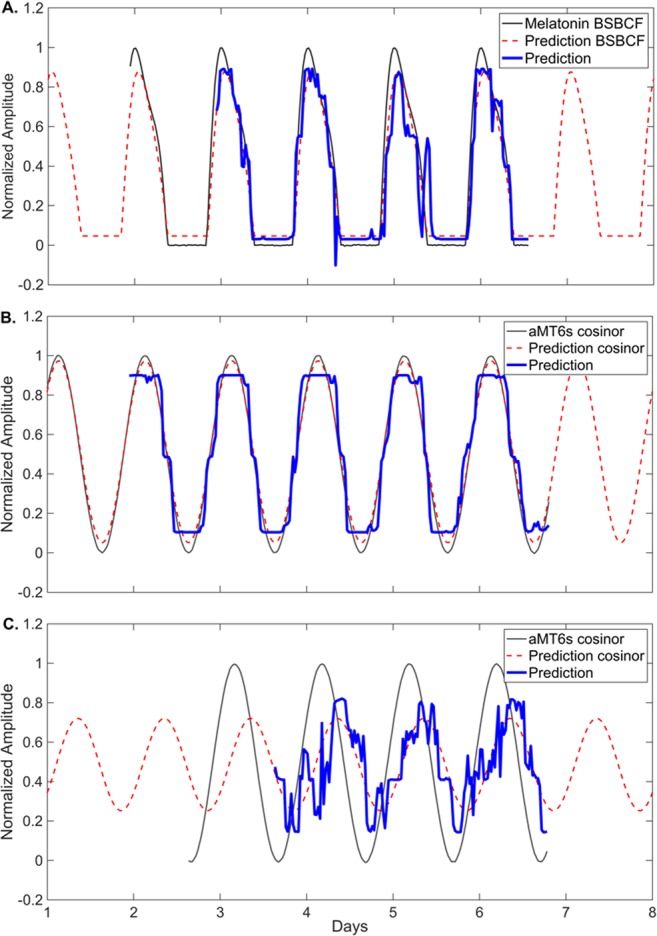
Example of network outputs using blue irradiance and wrist skin temperature inputs. (**A**) Predicted melatonin rhythm for one participant using the network trained on fixed sleep data. Prediction error is 11.69 minutes. (**B**) Predicted aMT6s rhythm for one participant using the non-shift work network trained on fixed and habitual sleep data. Prediction error is 4.52 minutes. (**C**) Predicted aMT6s rhythm for one participant on the night shift schedule, predicted using the SWnight network. Prediction error is 205.9 minutes. Black represents the fitted reference aMT6s waveform, blue represents the predicted aMT6s waveform, red represents the fitted cosine curve to predicted waveform.

A schematic of the neural network model is shown in Fig. 2A. The model consists of three layers: an input layer, one hidden layer with 5 neurons, and an output layer. All results utilize models trained using blue irradiance and skin temperature inputs, as these inputs have previously been reported as providing the most accurate circadian phase predictions^15^. Each model was trained using leave-one-out cross-validation with the same artificial neural network structure under four conditions: (i) fixed sleep (FS) using salivary melatonin data for the FS condition; (ii) fixed sleep (FS) and habitual sleep (HS) using urinary aMT6s data for FS and HS conditions; (iii) diurnal schedules using urinary aMT6s data for the FS, HS, and SWday conditions; and (iv) rotating shift work using urinary aMT6s for the SWnight condition. The model trained on diurnal schedules was also used to predict aMT6s data for the SWnight condition to assess the model’s generalizability to conditions with circadian misalignment. Different model inputs and their performances are summarized in Fig. 2B. Example model outputs from two well predicted (one melatonin, one aMT6s) and one poorly predicted participant are shown in Fig. 3.

Different combinations of input variables (light, skin temperature, activity) were trialed for each sleep-wake schedule (see Supplementary Table S2). For model performance across all input combinations see Supplementary Table S3. Overall, models trained with a combination of a light input and at least one skin temperature input tended to have highest accuracy, although no single combination performed consistently better across every trial.

### Fixed sleep

**Figure 4:**
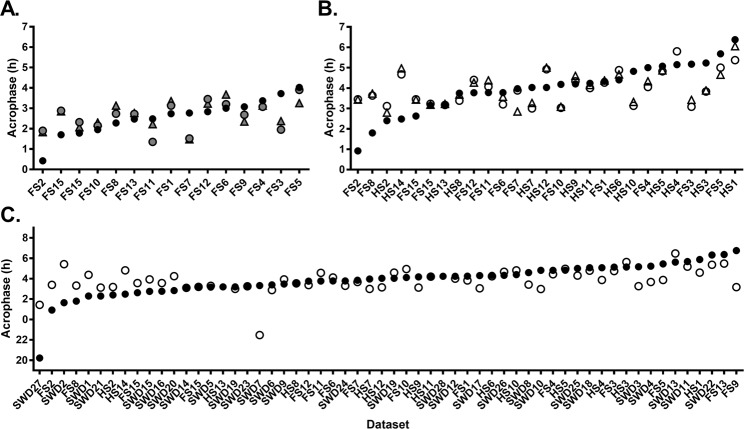
Measured circadian phase (black circles) and predicted circadian phase on diurnal schedules (using 11 temperature sensors in triangles; using one temperature sensor in circles) on diurnal schedules. (**A**) A network trained on fixed sleep data, with salivary melatonin reference phase (grey circles/triangles). (**B**) A network trained on both fixed (FS) and habitual (HS) sleep data, with urinary aMT6s reference phase (open circles/triangles). (**C**) A network trained on all diurnal sleep schedules (FS, HS, and day shift work schedules) with urinary aMT6s reference phase (open circles). Participants are ranked by reference circadian phase, from earliest to latest. Participants are labeled on x-axis based on schedule type and (arbitrary) numerical code.

The model was first trained and tested against the salivary melatonin data using blue light and 11 temperature sensors from the FS condition. This was a close replication of the original^15^ method and dataset. As shown in Fig. 4A, the model predicted salivary melatonin phase with a mean absolute error of 42.5 ± 25.2 minutes (mean error 5.7 ± 50.4 minutes), predicting 73% of individuals to within ±1 hour of measured phase and 100% of individuals to within ±2 hours.

To test whether the number of temperature sensors used as an input to the model could be reduced, we trained the same model with 1 temperature sensor (the non-dominant wrist) instead of 11. This had little impact on model accuracy, with a mean absolute error of 40.9 ± 32.0 minutes (mean error 1.3 ± 53.0 minutes), predicting 67% of individuals to within ±1 hour and 100% of individuals to within ±2 hours of measured phase, respectively. Predicted phase using a single or 11 skin temperature sensors were strongly correlated (*r = *0.86, *p* = 0.005, *n* = 15), with no significant difference in prediction error (*t*(14) = 0.75, *p* = 0.46).

**Figure 5:**
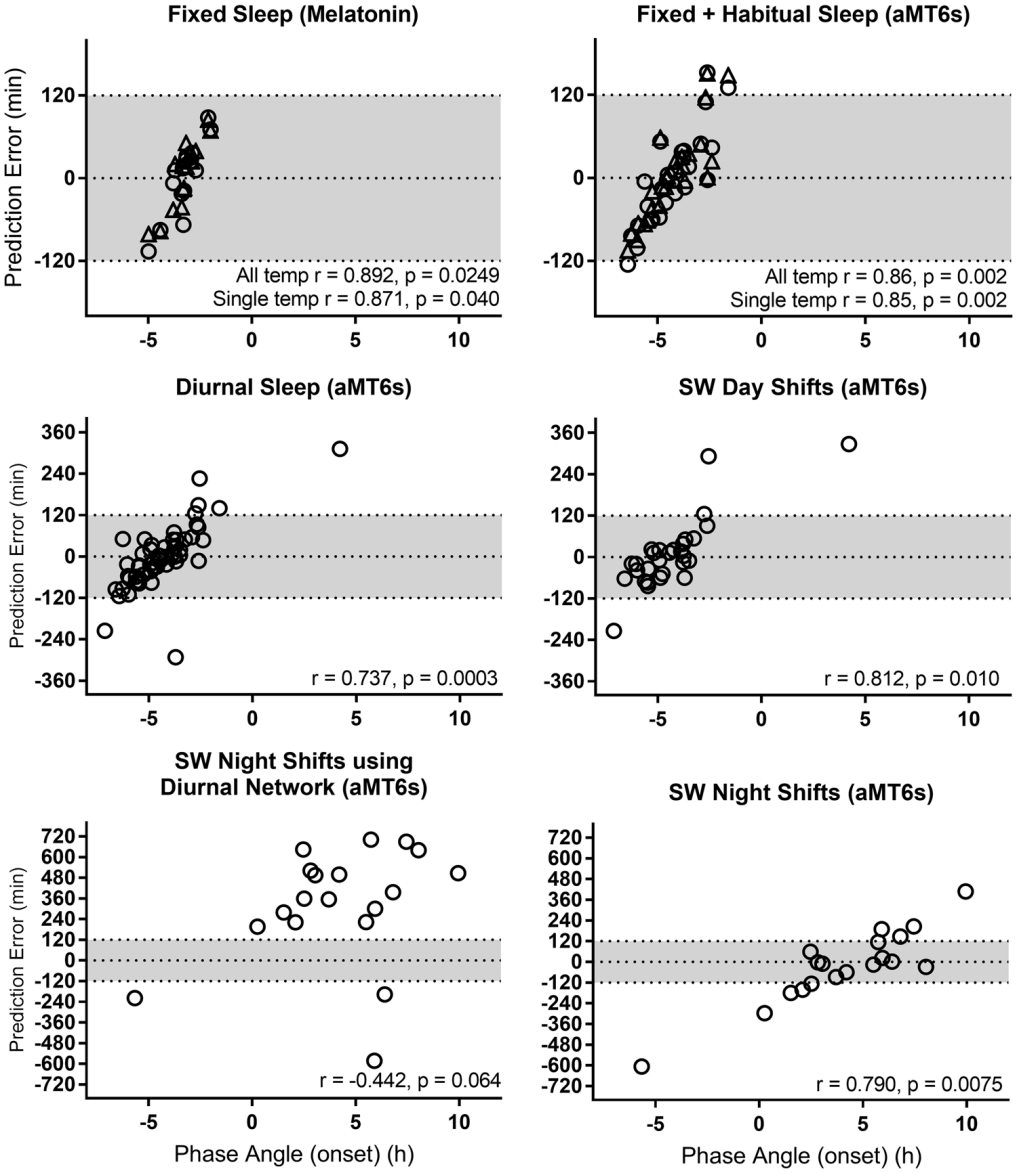
Relationship between prediction error and phase angle of entrainment, calculated as the difference between reference circadian phase and average sleep onset for each dataset. Networks trained with 11 temperature sensors are represented in triangles, and with 1 temperature sensor in circles. Grey shading indicates a ±2 h error window. Negative phase angle indicates sleep onset occurred before melatonin/aMT6s acrophase; positive phase angle indicates sleep onset occurred after melatonin/aMT6s acrophase. Correlation coefficients were calculated using circular statistics.

Prediction error had a significant relationship with phase angle of entrainment between melatonin phase and mean sleep onset (*r* = 0.89, *p* = 0.025; Fig. 5), such that predictions that were later than measured melatonin phase were strongly associated with a shorter phase angle (i.e., melatonin phase occurring closer to sleep onset). When using the single temperature sensor there was a modest relationship between prediction error and age (*r* = −0.52, *p* = 0.045). However, this relationship was non-significant (*p* = 0.18) after removing a single outlier who was aged 45 in the non-shift work study sample (mean age 26.0 ± 6.4 years, range 20–45). No relationship was found between prediction error and length of input data (*r* = −0.51, *p* = 0.054), or MEQ score (*r* = 0.22, *p* = 0.42).

### Fixed and habitual sleep

The model was next trained and tested against a combination of fixed (FS) and habitual (HS) schedules, using a more practical phase marker: urinary aMT6s. The model trained using 11 temperature sensors predicted urinary aMT6s acrophase with a mean absolute error of 48.7 ± 44.2 minutes (mean error 1.9 ± 66.5 minutes), predicting 65% of individuals to within ±1 hour and 92% to within ±2 hours, respectively (Fig. 4B). Predictions that were later than measured aMT6s acrophase were again strongly associated with a shorter phase angle (*r* = 0.86, *p* = 0.002; Fig. 5). No relationship was found between prediction error and length of input data (*r* = 0.06, *p* = 0.64), age (*r* = −0.32, *p* = 0.11), or MEQ score (*r* = 0.15, *p* = 0.45).

Similar to models trained on fixed sleep only, model predictions using 1 vs. 11 temperature sensors were highly correlated (*r* = 0.92, *p* < 0.001), with no significant difference in prediction error when using a single temperature sensor on the wrist (*t*(25) = −1.157, *p* = 0.26). Given these findings, we used a single temperature sensor (non-dominant wrist) and blue irradiance as inputs for the diurnal and shift work schedule models below.

### Diurnal sleep

The model was next trained on all the diurnal sleep schedules, which included FS, HS, and SWday (i.e., excluding night shifts). The model predicted aMT6s acrophase with a mean absolute error of 62.8 ± 67.1 minutes (mean error −1.0 ± 92.4 minutes), predicting 67% to within ± 1 hour and 89% to within ± 2 hours (Fig. 4C). When using the diurnal model, there were no significant differences in absolute prediction error between the FS + HS (50.5 ± 41.1 min) and SWday (74.3 ± 83.7 min) datasets (*t*(54) = 1.33, *p* = 0.19). Predictions for non-shift work datasets (FS + HS) using the model trained on all diurnal data were also highly correlated with predictions generated by the model trained using only the non-shift work data presented above (*r* = 0.96, *p* < 0.001).

Prediction error had a significant relationship with phase angle of entrainment between aMT6s acrophase and mean sleep onset (*r* = 0.74, *p* < 0.0003; Fig. 5), such that predictions that were later than measured aMT6s acrophase were strongly associated with a shorter (or positive) phase angle (i.e., aMT6s acrophase occurring closer to sleep onset). There was no relationship between prediction error and length of input data (*r* = 0.06, *p* = 0.64), age (*r* = −0.10, *p* = 0.49), or MEQ score (*r* = −0.11, *p* = 0.40).

### Night shift work

To evaluate whether the model could generalize to predict circadian phase during night shift work, we first tested whether the model trained on the diurnal schedules could accurately predict aMT6s acrophase on the night shift schedule (SWnight). Performance was poor across all metrics (Table 2). The mean absolute error was 422.6 ± 170.2 minutes (mean error 317.7 ± 337.0 minutes), with no individuals predicted within ±2 hours of measured acrophase time. Prediction error was significantly larger on the SWnight schedule compared to the diurnal datasets (*t*(73) = 6.468, *p* < 0.0001). Prediction error on the SWnight schedule was not associated with phase angle (*r* = −0.44, *p* = 0.064), age (*r* = 0.22, *p* = 0.37), MEQ score (*r* = 0.10, *p* = 0.69), phase shift from SWday to SWnight (*r* = −0.15, *p* = 0.55), or length of dataset (*r* = −0.09, *p* = 0.72).

**Table 2:** Neural network prediction error summary

Network	# skin temp inputs	Reference rhythm	Prediction method	n	Prediction Error (minutes)				Prediction Error Absolute Values (minutes)						Correlation with measured phase		Percentage predicted within range			
					Mean	Median	SD	Range	Mean Absolute	Median Absolute	SD Absolute	Min	Max	RMSE	r	p	± 15 mins	± 30 mins	± 60 mins	± 120 mins
***Trained networks***																				
Fixed sleep	11	Melatonin	cross validation	15	5.67	17.82	50.36	166.03	42.49	39.43	25.24	14.21	84.91	48.98	0.43	0.12	6.67	40.00	73.33	100.00
Fixed sleep	1	Melatonin	cross validation	15	1.33	11.48	53.00	194.61	40.86	27.01	31.97	7.06	106.29	51.23	0.41	0.15	26.67	53.33	66.67	100.00
Fixed + habitual sleep	11	aMT6s	cross validation	26	1.88	−1.46	66.46	256.29	48.71	37.76	44.19	0.81	151.32	65.20	0.46	0.07	26.92	42.31	65.38	92.31
Fixed + habitual sleep	1	aMT6s	cross validation	27	−0.91	−4.16	66.83	277.90	50.85	40.98	42.22	2.74	152.60	65.59	0.45	0.03	22.22	37.04	66.67	88.89
Diurnal sleep	1	aMT6s	cross validation	56	−0.95	−3.27	92.36	604.60	62.84	48.60	67.16	0.42	312.66	91.54	0.42	0.01	19.64	41.07	66.07	87.50
SWday	1	aMT6s	cross validation	29	9.36	−9.21	104.08	541.49	65.68	38.28	80.34	3.34	327.79	102.69	0.39	0.16	17.24	41.38	68.97	86.21
SWnights	1	aMT6s	cross validation	19	−22.11	−10.24	212.86	1013.81	143.29	115.28	155.42	1.50	605.48	208.36	0.04	0.84	21.05	31.58	42.11	52.63
***Predictions using final networks***																				
SWday	1	aMT6s	Fixed + habitual sleep	29	−2.85	−0.41	145.69	884.74	81.87	50.11	119.55	0.41	561.90	143.18	0.46	0.08	24.14	37.93	68.97	82.76
SWnight	1	aMT6s	Fixed + habitual sleep	19	314.26	375.79	355.86	1245.97	437.37	403.83	170.18	198.53	695.89	467.69	0.23	0.36	0.00	0.00	0.00	0.00
SWnight	1	aMT6s	Diurnal sleep	19	317.70	358.75	337.00	1284.05	422.58	396.71	177.79	196.85	701.92	456.64	0.31	0.22	0.00	0.00	0.00	0.00
***Comparative estimation methods***																				
Diurnal sleep	-	aMT6s	mid-sleep	29	−29.45	−43.44	104.19	790.70	73.20	79.27	60.75	0.50	542.90	107.38	0.25	0.17	14.29	26.79	50.00	91.07
SWnight	-	aMT6s	mid-sleep	19	413.13	436.30	185.67	853.40	427.17	436.30	148.38	133.40	720.00	450.92	0.34	0.09	0.00	0.00	0.00	0.00
Diurnal sleep	-	aMT6s	normal range estimate	29	11.05	3.31	150.15	807.18	115.15	100.43	95.76	1.40	542.90	174.66	−0.04	0.75	8.93	14.29	33.93	57.14
SWnight	-	aMT6s	normal range estimate	19	−55.73	−52.96	206.87	854.11	150.01	96.40	149.40	4.42	545.78	208.92	0.12	0.57	5.26	5.26	26.32	63.16
Diurnal sleep	-	aMT6s	random re-sampling	29	0.00	0.00	130.92	900.00	95.71	88.39	87.50	0.00	469.00	129.75	0.10	0.43	8.93	19.64	30.36	64.29
SWnight	-	aMT6s	random re-sampling	19	0.00	11.00	235.99	1012.00	180.11	146.46	148.00	11.00	547.00	229.70	0.33	0.10	5.26	10.53	21.05	36.84

Note: all results are from networks using inputs of blue light irradiance combined with skin temperature. See Supplementary Table S3 for results using alternate input combinations. aMT6s = urinary 6-sulphatoxymelatonin, RMSE = root mean square error. Correlation coefficients were calculated using circular statistics.

**Figure 6:**
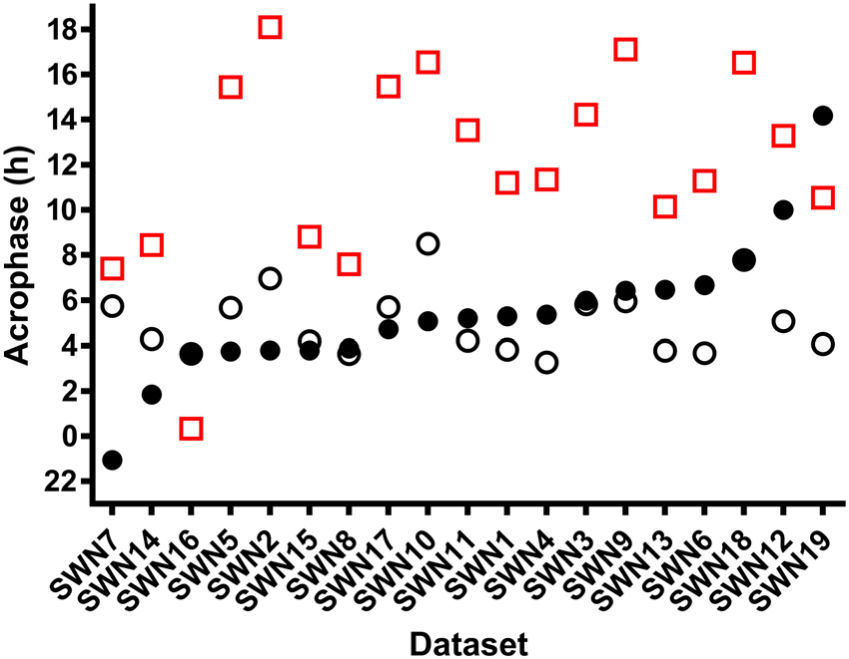
Measured and predicted urinary aMT6s acrophase after multiple consecutive night shifts. Urinary aMT6s acrophase is shown as filled black circles. Predicted acrophase using a network trained on all diurnal datasets is shown as red squares, and a network trained on night shift work schedules only is shown as open black circles. Participants are ranked by reference circadian phase (aMT6s), from earliest to latest. Participants are labeled on x-axis based on schedule type and (arbitrary) numerical code.

When trained using only data from the night shift schedule, prediction accuracy improved compared to using the diurnal model (Fig. 6), but prediction error remained large; mean absolute error was 143.3 ± 155.4 minutes (mean error −22.1 ± 212.9 minutes), with 42% predicted to within ±1 hour and 53% to within ±2 hours. Prediction error was significantly associated with phase angle (*r* = 0.79, *p* = 0.008; Fig. 5), such that predictions that were later than measured aMT6s acrophase were strongly associated with a more positive phase angle (i.e., aMT6s acrophase occurring earlier relative to sleep onset). Greater eveningness scores were also associated with larger prediction errors (predictions later than aMT6s acrophase; *r* = 0.55, *p* = 0.02). Prediction error was not associated with age (*r* = 0.45, *p* = 0.051), phase shift from SWday to SWnight (*r* = 0.31, *p* = 0.20), or length of dataset (*r =* −0.24, *p* = 0.32).

### Comparative performance of model vs. simple phase estimates

To assess whether predictions of circadian phase using the neural network models were improved relative to a simple sleep-based estimate, average mid-sleep time was used as a substitute for aMT6s acrophase time (Table 2). On the diurnal schedules, there was no significant difference in mean absolute error between mid-sleep time (73.2 ± 79.3 minutes) compared to the model trained on diurnal data (62.8 ± 67.2 minutes; *t*(55) = −1.21, *p* = 0.23). On the night shift schedule, mid-sleep time was a poor proxy for aMT6s acrophase time, with consistently worse performance compared to the model trained on the night shift schedule data. Mean absolute prediction error using mid-sleep time (427.5 ± 149.0 minutes) was significantly larger compared to model predictions on the night shift schedule (143.3 ± 155.4 minutes; *t*(18) = 5.25, *p* < 0.001).

Additionally, to determine whether model predictions performed better than chance, we simulated “guessing” circadian phase based on the reported range of urinary aMT6s acrophase in sighted individuals^27^. Selecting a clock time at random between 1.3 and 7.1 h for each dataset resulted in a mean absolute error of 115.2 ± 95.8 minutes on the diurnal schedules, and 150.0 ± 149.4 minutes on the night shift schedule (Table 2). Relative to this benchmark, all models performed better than an educated guess, except for predictions made on the night shift schedule using models trained on diurnal data or using mid-sleep time as a proxy on the night shift schedule.

## Discussion

In this study we sought to determine whether a previously published artificial neural network model would generalize to predict circadian phase on a range of sleep-wake schedules using continuous ambulatory recordings of light and skin temperature data. We found that the model, using inputs of blue light irradiance (measured from the wrist) and skin temperature (measured from distal and proximal sensors), predicted salivary melatonin phase with mean absolute error < 43 minutes in individuals living on a stable diurnal sleep schedule, and that a single wrist-worn temperature sensor, combined with blue light irradiance, can be used without compromising prediction accuracy. We further demonstrated that the model can be trained to predict urinary aMT6s acrophase with mean absolute error < 64 minutes in both healthy individuals on a diurnal schedule and rotating shift workers on a diurnal shift schedule. In its current form, however, the model did not extend to predict circadian phase in shift workers on a night shift schedule (mean absolute error 423 minutes), although model performance improved when trained solely on night shift data (mean absolute error 143 minutes).

Our findings are an important replication of a model developed by Kolodyazhniy, *et al*.^15^, which showed remarkably precise predictive ability when applied to data collected over a stable sleep-wake schedule in a group of healthy young male participants. Our model performance is consistent with that of Kolodyazhniy, *et al*.^15^ on a comparable sleep-wake schedule, even when including female participants and using a wrist-worn light sensor, which is known to be less accurate in estimating retinal light exposure compared to eye-level sensors^28^ of the kind used by Kolodyazhniy *et al*.^15^. We extended the approach to more variable real-world sleep scenarios, generalizing the neural network approach to a broader range of demographics (e.g., females, age range, medical history), and sleep-wake patterns by including a shift work population with highly variable circadian phase relationships with sleep. Furthermore, we found that models based on wrist temperature provide similar results to models using inputs from 11 skin temperature sensors. This finding is promising for future development of wearable devices to monitor circadian timing with low participant burden, although further work is required to determine the conditions under which a single sensor is a valid substitute for multiple sensors.

The neural network model’s accuracy on diurnal schedules (mean absolute error < 64 minutes) was comparable to other reported methods that used wrist temperature, light, and body position (~46 minutes^13^); light and heart rate in an autoregressive model (~36 minutes^12^); and light and activity in a limit-cycle oscillator model (~41–87 minutes^16,17^). In theory, the combination of a physiological measurement (e.g., skin temperature, heart rate) with a measurable endogenous rhythm, and an environmental time cue (light) would enable models to more accurately predict phase. We obtained similar results using light inputs alone, however, indicating that the additional skin temperature inputs may have a relatively modest contribution to predictions. This may be because skin temperature is masked by other factors, and therefore not reflective of the endogenous circadian pacemaker. Consistent with this interpretation, we found models that included only skin temperature inputs consistently performed worse than models that included light inputs, particularly in the shift work datasets (see Supplementary Table S3).

One of our key findings is that the model can be trained to predict urinary aMT6s acrophase, a commonly used marker of circadian timing in field conditions^23–25,29–35^. This is important, since urine can be sampled by individuals in normal living conditions without the disruption to daily activity/sleep required for a dim light melatonin onset (DLMO) or full melatonin profile assessment in the laboratory. While we are not aware of any direct validation of urinary aMT6s against plasma/saliva melatonin in a shift work population, studies have consistently reported a strong correlation between the timing of urinary aMT6s and salivary and/or plasma melatonin phase within an individual^36,37^, and a consistent timing offset of between 1.5 and 2 hours, both in laboratory^11,38^ and field conditions^39^. Furthermore, similar phase shifts in salivary and plasma melatonin and urinary aMT6s have been observed in response to light^40^, indicating that these phase relationships remain stable across dynamic changes in circadian timing. This is to be expected because aMT6s is the main metabolite of melatonin, and the melatonin rhythm is under direct control by the master circadian clock in the SCN^11^. Nevertheless, we acknowledge that the use of a different phase marker is a potential source of additional error in the night shift condition.

A key limitation of the models presented here is the inability to predict the observed population range in circadian phase, even under diurnal conditions. Results reported by Kolodyazhniy, *et al*.^15^ were striking in their prediction of individual-level phase, with a strong positive relationship between predicted and measured melatonin phase (*r* = 0.96). For each model we trained, however, we found limitations replicating the observed range in circadian phase. Similar problems predicting the full range of measured phases have been reported using alternate mathematical modelling approaches for predicting circadian phase in field settings, including autoregressive^12^ and limit-cycle oscillator models^16,17^. Our findings indicate that the model is least accurate for individuals with extreme circadian timing. Given the known inter-individual variability in circadian phase, both in individuals on stable sleep-wake schedules^4,41,42^, as well as in shift workers^24–26,31^, it is important that a model be able to estimate individual-level timing before it is used in applied settings (e.g., for designing experimental procedures or treatments for circadian rhythm disorders).

A possible reason for the limited prediction of the population range of melatonin timing may be the use of a reference waveform based on a 30–48 hour melatonin/aMT6s assessment. The models were trained using multiple days of input data, assuming either a stable phase position (non-shift work datasets and first aMT6s assessment in shift work datasets *n* = 51), or assuming a linear daily phase change between two phase assessments (shift work datasets n = 26). Day-to-day changes in circadian phase have not been well characterized in normal living conditions. In healthy individuals on a regular sleep schedule, a difference in DLMO of approximately 45 minutes has been reported when measured a week apart^43^. In the few studies that have continuous circadian phase measurements, via urinary aMT6s, over a change in light-dark cycle; e.g., shift work^24^ and simulated jetlag^44^, there are individual differences in the rate of phase change. Differences in light patterns likely underlie these inter-individual differences, as shown in shift workers^25,31^, along with physiological differences such as light sensitivity^45,46^. Therefore, there are likely to be inter-daily changes in phase that could not be accounted for when training the model. Daily phase measurements would likely improve model predictions, allowing the model to learn subtle phase relationships with irregular light/sleep patterns over multiple days.

Estimating circadian phase when sleep-wake patterns are decoupled from the endogenous circadian pacemaker, such as in most night shift workers^22^, is an especially challenging test of the model. We found that the model trained on ‘diurnal’ data made universally poor predictions of aMT6s acrophase on the night shift schedule. Similarly, estimation of aMT6s acrophase using mid-sleep time was worse than chance on the night shift schedule. In most cases, both mid-sleep time and the model predicted a circadian phase indicative of re-alignment between circadian and work-rest rhythms, with predicted phase occurring during daytime rest. However, only two participants showed evidence of circadian adaptation to the night shift schedule (aMT6s acrophase occurring within the sleep episode). This finding suggests that a model trained on data where sleep-wake and melatonin phase are reasonably aligned will not generalize to night shift workers. Model performance did improve for at least half the individuals when trained separately on the night shift schedule data (52% predicted within ±2 hours), though errors remained large. This result experimentally supports the supposition by Kolodyazhniy *et al*.^15^ that phase may not be reliably predicted in circumstances where the timing of sleep and activity are drifting such as in rotating shift work. Notably, the neural network model performed worse than limit cycle oscillator models used to predict circadian phase under night shift conditions^47,48^.

Our findings suggest that larger datasets may be needed to adequately train a neural network model to make predictions in a noisy/complex scenario such as seen in night shift workers. The predicted rhythm amplitude was markedly lower on the night shift datasets, especially for individuals with large prediction errors (e.g., Fig. 4B), perhaps reflecting irregular or lower amplitude rhythms in the light and skin temperature inputs. As model parameters are data-driven, provision of a wide range of phase relationships is important for the model to learn the complex phase relationships between endogenous circadian rhythms and behavior. Additional structural adjustments to the network (e.g., additional hidden layers) may also be needed to properly account for the complex phase dynamics in circumstances of circadian misalignment with sleep. Due to the larger number of model parameters, testing of more complicated network structures would also require more data than available in the current dataset. Alternative approaches, such as the use of biomarkers to predict circadian phase^49–51^, should also be trialed in a shift work setting.

A potential limitation of this study is the use of a wrist-worn light sensor, which is a less accurate measure of retinal light exposure compared to eye- or shoulder-level sensors^28^. This may account for the larger mean prediction errors observed compared to Kolodyazhniy *et al*.^15^, who used eye-level light sensors. Nevertheless, validation using wrist-worn sensors is important given their widespread use and practicality in a range of field settings. In order to replicate the Kolodyazhniy, *et al*.^15^ model’s inputs, and due to the known sensitivity of the circadian pacemaker to blue light^52,53^, we used the blue irradiance variable from the Actiwatch Spectrum. We observed only small differences in model accuracy when using photopic illuminance (white lux) or blue irradiance variables (see Table S3), although this may be because the white lux output is composed of a linear combination of the RGB sensors^54^. Using a subset of our participants, we confirmed that there is a strong correlation between the white lux and blue irradiance data (*n* = 8, mean ± SD Person’s *r* = 0.97 ± 0.01, *p* < 0.0001). Future work using devices capable of measuring melanopic lux (480 nm^55^) may improve predictions of the biological responses to light exposure.

Additionally, while we controlled for menstrual phase in female participants in the non-shift work study, we did not control for menstrual phase or use of hormonal contraception in the shift work study. Both menstrual phase and use of oral contraceptives are known to influence the amplitude of melatonin and core body temperature rhythms^56–58^. The aim of this study was, however, to test the model’s generalizability to *real-world* conditions: under these conditions a model would need to be useful regardless of sex or contraception use.

## Conclusions

In summary, the neural network model shows promise for estimation of circadian phase in real world but stable conditions, including during day/evening shifts in rotating shift workers. The approach requires easily collected ambulatory light and wrist temperature data, and can be applied to urinary aMT6s acrophase as well as salivary melatonin predictions. Non-invasive circadian phase prediction has broad clinical and research applications, including ongoing phase monitoring in normal living conditions. At this stage, the model has difficulty simulating individual variation in circadian phase, and is not currently suitable for use in cases of circadian misalignment, such as observed in night shift workers. Modifications to the network structure, and training using larger datasets may improve model accuracy.

## Methods

### Protocol

Data were collected during two studies: In healthy regular diurnal sleepers (the *non-shift work study*), and in rotating shift workers working in an Intensive Care Unit (the *shift work study*). For the non-shift work protocol, participants completed their usual daily activities for approximately one week, with no set bed or wake times (*habitual sleep; HS*), followed by two weeks of a structured 8:16 h sleep:wake schedule, with fixed sleep and wake times based on reported habitual sleep timing during week one (*fixed sleep; FS*). During this time participants recorded their sleep and wake times in daily sleep diaries, made scheduled telephone call-ins to a study phone to log their bed and wake times, and wore a wrist actigraph device to record activity-rest patterns. Additionally, skin temperature from 11 skin sites was measured with small wireless recording devices (iButtons). Ambient light was recorded using the light sensor built into the wrist actigraph device (irradiance in mW/cm^2^, wavelength range 400–700 nm, Actiwatch Spectrum, Philips Respironics, Bend, OR, USA). Participants were required to refrain from consuming alcohol and caffeine throughout the protocol. After the three weeks of ambulatory monitoring, participants stayed in a laboratory suite free of time cues at the Monash Sleep and Circadian Rhythm Laboratory, as part of a broader protocol described elsewhere^59^. Participants were admitted to the laboratory on a Monday afternoon, and spent two baseline days with habitual bed times, followed by a 40-hour constant routine protocol.

Circadian rhythms were measured using salivary melatonin, and urinary 6-sulphatoxymelatonin (aMT6s). Salivary melatonin was measured in hourly samples during a 40-hour constant routine in the laboratory. Urinary aMT6s was measured during two 48-hour urine collection periods timed to occur at the end of the HS monitoring, and at the end of the FS recording, which was over the two baseline days in the laboratory.

For the shift work study nursing and medical staff were monitored as they worked a rotating shift schedule in the Intensive Care Unit at Austin Health, Heidelberg, Australia. Shift schedules consisted of an irregular series of day or evening shifts or days off (*SWday*), followed by at least three consecutive night shifts (*SWnight*). Nurse rosters included a variable pattern of day (07:00–15:30 h), evening (15:00–21:30 h) and night (21:00–07:30 h) shifts, while medical staff rosters consistently rotated through 7 consecutive day shifts (08:00–20:30 h), 7 days off, and 7 consecutive night shifts (20:00–08:30 h). Participants recorded their sleep-wake and work times in a daily log, and wore a wrist actigraph (same model as in the non-shift work study) to record activity-rest patterns and ambient light levels. To minimize participant burden, skin temperature was recorded on a single skin site only (non-dominant wrist). Due to the field conditions, there was limited control over the number of days that participants could be monitored on each type of shift. The circadian marker in this protocol was urinary aMT6s. Participants completed three 24–48 h urine collections, timed to occur on the last rostered day shift, on their first night shift (night 1), and during their final consecutive night shift.

### Participants

#### Non-shift work study

Sixteen participants (3 female) completed the study. Healthy participants aged 20 to 45 years (26.45 ± 6.41, mean ± SD), who lived on a normally entrained 24-h schedule were included. Participants had a range of evening to intermediate morningness-eveningness scores (38.75 ± 3.38, range 34–45) and normal body mass index (BMI; 22.64 ± 2.44, range 19.20–28.22 kg/m^2^). Participants had no self-reported history of medical, psychiatric, or sleep disorders, and had not traveled across three or more time zones in the last month, or been engaged in shift work (5 or more hours worked between 22:00 and 07:00 h) in the last three months. Participants were excluded if they reported use of illicit drugs within the last year, consumption of more than 300 mg/day of caffeine, or 14 standard units/week of alcohol. Female participants were recruited if they were not using hormonal contraception or pregnant. Menstrual phase was monitored by self-report, with female participants admitted to the laboratory at the commencement of the follicular phase.

#### Shift work study

Twenty-eight participants (22 nurses [18 F], 6 doctors [3 F]) completed the shift work study. Participants were aged 23 to 64 years (33.14 ± 9.72), with a range of extreme evening to intermediate morningness-eveningness scores (38.11 ± 7.10, range 23–52) and a range in BMI (24.46 ± 3.92, range 15.19–32 kg/m^2^). There were no exclusion criteria or limitations on alcohol or caffeine consumption or medication use.

#### Study approval

All procedures for both protocols were approved by the Monash University Human Research Ethics Committee and were performed in compliance with standards set by the latest revision of the Declaration of Helsinki. Procedures for the shift work protocol were also approved by the Austin Health Research Ethics Committee. Participants provided written informed consent, and received financial compensation at the completion of their participation.

### Ambulatory measurements

Participants wore a wrist actigraph (Actiwatch Spectrum, Philips Respironics, Bend, OR, USA) on the non-dominant wrist to monitor light exposure (red, green, and blue [RGB] irradiances, and estimated white lux) and activity at 1-minute epochs (medium sensitivity; 40 activity counts per epoch). Prior assessment of the spectral sensitivity of the Actiwatch Spectrum indicated a peak response wavelength of 460 nm for the blue sensor, 500 nm for the green sensor, and 655 nm for the red sensor^54^. Participants were asked to wear the device with the sensor uncovered, except when the device could be damaged (e.g., while showering, during contact sports), or when required due to operational demands (e.g., medical procedures conducted by participants during the shift work protocol).

Skin temperature was monitored using small wireless temperature recording devices (DS1922L Thermochron iButtons, accuracy 0.0625 °C, Maxim, San Jose, CA, USA) set to a 2.5-minute sampling interval. On the non-shift work protocol, iButtons were worn over 11 skin sites, in the configuration described in^15^: one on the left and right shoulders, wrists, thighs, calves and ankles, and one on the sternum. The iButtons were applied by a researcher at the start of the protocol and worn continuously for 7 days over the HS schedule. Due to the limited memory of the devices an additional set was provided for the 7 days of FS immediately prior to admission to the laboratory. Participants applied the second set of labeled iButtons at home and the location of each sensor was checked upon admission to the laboratory.

During the shift work protocol, one iButton was worn continuously over the protocol over the radial artery of the non-dominant wrist, alongside the Actiwatch. The wrist was selected due to the previously described endogenous rhythm in wrist temperature^13,60,61^, practicality within the operational setting, and potential for future device development. Due to the limited memory capacity the devices were downloaded and reset by a researcher at least once every 7 days. iButtons were held on the skin using medical tape. Each participant was provided tape with instructions to replace the tape as required to ensure constant sensor contact with the skin.

During the non-shift work protocol, participants kept a log detailing all times skin temperature monitors were removed, which was used for data cleaning (detailed below). No log was maintained during the shift work study.

### Melatonin assessments

#### Melatonin

Salivary melatonin was assessed on the non-shift work protocol via hourly saliva samples collected during a 40-hour constant routine protocol^62,63^. Participants maintained a semi-recumbent posture in dim light ( < 3 lux) and consumed hourly equicaloric snacks. No food or water was permitted in the 30 minutes prior to each sample. Participants were allowed 5 minutes to collect saliva via passive drool into a 10 mL sampling tube. Samples were aliquoted into 2 mL cyrovials in a biosafety cabinet before being snap frozen in dry ice, and stored at −20 °C. Saliva was assayed in duplicate (200 µL per duplicate) for melatonin via double antibody radioummunoassay, with standards and reagents supplied by Buhlmann Laboratories (RKDSM-2, Buhlmann Laboratories AG, Schönenbuch, Switzerland). The minimum detectable threshold was 4.3 pg/mL.

#### aMT6s

The urinary melatonin metabolite, 6-sulphatoxymelatonin, (aMT6s) was used as a marker of circadian phase in ambulatory conditions^27,36^. This marker was selected as it is widely used in field settings, particularly where salivary melatonin assessments are impractical, such as in shift work (e.g.,^23–25,29–31,35^). Urinary aMT6s acrophase has been validated against both plasma and salivary melatonin in controlled laboratory conditions^36,37^ and in field conditions^39^. Similar phase shifts for salivary and plasma melatonin, and urinary aMT6s have been shown in response to light in the laboratory^40^, indicating that these phase relationships remain stable across dynamic changes in circadian timing, as may be experienced by rotating shift workers. For each assessment, participants collected urine over 24–48 hours, in 4 hourly (8 hours over sleep) sequential collection blocks as previously described^26^. Samples were retained for each block in 5-mL cryovials and stored at −20 °C. The volumes and times of collections were recorded. Urinary aMT6s concentration was determined using radioimmunoassay^64^, using standards and reagents supplied by Stockgrand Ltd (aMT6s-HU-K200, Stockgrand Ltd., Guildford, Surrey, UK). The minimum detectable threshold was 0.5 ng/mL. See supplementary material for assay details.

### Data retention

Sixteen participants had eligible non-shift work protocol datasets; of these, 13 (81%) contributed datasets to both FS and HS analyses, two (13%) contributed a HS dataset only, and one (6%) a FS dataset only. One participant did not complete the FS component of the protocol, three participants were excluded due to poor quality aMT6s rhythms (two FS, one HS), one participant was excluded from training HS models due to insufficient light data, and another was excluded from training HS models on all skin temperature sensors due to a missing sternum recording.

Of the 28 participants on the shift work protocol, 11 contributed one dataset (73% one diurnal; 27% one night shift schedule), 14 contributed two datasets (93% one diurnal and one night shift schedule; 7% two diurnal) and three contributed three datasets (100% two diurnal and one night shift schedule), for a total of 48 individual datasets. Twenty-nine datasets were included over the diurnal shift schedule, with urinary aMT6s measured either over a day/evening shift schedule (n = 19) or including the first night shift (n = 10). Nineteen datasets were included over the night shift schedule, with urinary aMT6s reference phase measured after 3.95 ± 1.13 consecutive night shifts (range 3–7 nights).

The number of days included for input variables varied for each dataset (Table 1). Dataset length was 5.63 ± 0.87 days, (Mean ± SD, range 3.02–7.60) on the non-shift work study and 4.08 ± 1.59 days (Mean ± SD, range 1.56–8.90) for the shift work study. Data availability for each dataset is summarized in Supplementary Table 1. Prior to data processing, 96.5 ± 5.8% of light data were available pre-cleaning, and 85.0 ± 8.7% post-cleaning. Skin temperature data were available for 97.1 ± 8.4% pre-cleaning, and for 88.0 ± 11.8% post-cleaning.

### Data processing

All data processing was completed using MATLAB R2016b (The MathWorks, Natick, MA, USA), except for manual skin temperature data cleaning, completed using a combination of visual inspection and participant device removal log, and determination of sleep and wake times for light data cleaning, completed using a combination of sleep diaries and Actiware software, as previously described^25^. In order to replicate the neural network model approach, data processing steps followed those reported by Kolodyazhniy, *et al*.^15^, except where modifications were required to appropriately handle the shift work datasets (described below).

#### Determination of datasets

Data were split and processed in the following datasets determined by their sleep-wake schedule: (i) fixed sleep (FS); (ii) habitual sleep (HS); (iii) diurnal shift work schedule (SWday); and (iv) night shift work schedule (SWnight). Data from individuals on the non-shift work protocol were used for datasets (i) and (ii), and data from individuals on the shift work protocol were used for datasets (iii) and (iv). Each participant’s data were processed individually, but could be included in multiple datasets.

For the non-shift work protocol, data were split into HS (all ambulatory data on the habitual sleep schedule up to the final urine sample of the at-home circadian rhythm assessment), and FS (ambulatory data over the final week of structured-sleep, up to the end of the second baseline day in the laboratory). For the shift work protocol, datasets were split based on the timing of the circadian rhythm assessments. Due to a similar shift schedule over the ambulatory recording, and a lack of significant circadian phase changes on first night shift, datasets prior to a day shift or first night shift circadian phase assessment were grouped as ‘diurnal’ shift work datasets for analysis purposes. Consequently, SWday included ambulatory data up to the end of the day/evening shift, and/or first night shift urine collection; SWnight included ambulatory data from the first night shift to end of the final night shift urine collection.

#### Skin temperature

Temperature data were processed following the same steps as reported by Kolodyazhniy, *et al*.^15^, using all 11 sensors, or a single wrist sensor worn on the non-dominant wrist. In the case of 11 sensors, data from the right- and left-hand side of each skin site were averaged to create 6 variables; one for the shoulders, wrists, thighs, calves, and feet, plus the single sternum sensor. The moving average for each variable (or single sensor) was calculated by averaging data in the interval of ±12 h from the center of the moving window. Data were detrended by subtracting the moving average with a 24-h window from each temperature variable, and then scaled using z-transformation. Values outside of ±2 standard deviations were considered outliers and removed. Missing data were interpolated (see below).

Prior to processing, artefacts due to device removal were removed using the following steps. For the non-shift work dataset, data were excluded where the participant logged device removal, or where the pattern of recording from all 11 sensors consistently showed a sustained rapid drop in temperature, determined by visual inspection. For the shift work dataset, where only one skin temperature sensor was worn on the non-dominant wrist, it was assumed that whenever the actigraph device was removed, the iButton was also removed, due to operational requirements of the hospital. Thus, skin temperature data were treated as missing where the actigraph ‘off-wrist’ sensor was activated. This assumption was checked via visual inspection of the two variables. Further data cleaning was implemented over all skin temperature data (both datasets): values below 20 °C were removed and outliers > 3 standard deviations from the mean of that variable were excluded.

#### Light

Light data were processed using the same approach as Kolodyazhniy, *et al*.^15^, whereby irradiance values < 0.01 lux were replaced with 0.01 lux to allow log transformation. All light data streams were then log_10_-transformed.

Due to use of a different light measurement device to that used by Kolodyazhniy, *et al*.^15^ we developed additional cleaning steps, which were implemented prior to processing. Data were excluded when Actiware software determined the device was not being worn or during wake times with values of <1 lux, which were considered artefacts due to coverage of the light sensor by clothing^65^. Missing values were interpolated (see below).

#### Activity

Activity counts were extracted from wrist actigraph devices, and were excluded when Actiware software determined the device was not being worn. Missing data were interpolated (see below). Values <0 were replaced with 0 to remove interpolation artefacts. Subsequently, processing steps described in Kolodyazhniy, *et al*.^15^ were implemented: data were scaled using z-transformation and outliers (>2 standard deviations) were removed.

#### Missing data

Prior to processing, all data streams were required to meet three inclusion criteria: (i) <50% missing data for all variables; (ii) a minimum of 3 days’ total length of recording, including missing data; and (iii) no gaps greater than 36 hours, or greater than 3 hours within the first day of the recording. A summary of available data for included datasets is shown in Supplementary Table S1.

Interpolation was completed using the MATLAB function ‘fillgaps’, which uses autoregressive modeling to interpolate missing data. Interpolation was applied with the following rules: if missing data occurred over a period of ≥3 hours within the first day of the recording, data were discarded up to the end of the missing period. If the missing data occurred over a period of ≤1 hour, data were interpolated using default ‘fillgaps’ settings. All other periods of missing data were interpolated with the ‘fillgaps’ function using data recorded one day prior to the missing data, to an order of half a day [i.e., fillgaps(data up to end of gap, length(1 day before gap), length(12 hours))]. This method was used for interpolation of missing data in both light, activity and temperature.

#### Reference circadian phase

*Melatonin:* The melatonin reference waveform was based on salivary melatonin levels measured from 38 hourly samples in CR conditions for each participant. Melatonin data were processed following the same procedures reported in Kolodyazhniy, *et al*.^15^, with data resampled to 1 minute epochs using linear interpolation and the time vector converted to radians (2π = 24 h). Data were then fit to a Bimodal Skewed Baseline Cosine Function (BSBCF)^66^ using a non-linear least squares curve fitting method, assuming a period of 24 h:

$$\begin{array}{c}f(t)=b+\frac{H}{2(1-c)}[(\cos (t-\varphi +v\,\cos (t-\varphi ))+\,m\,\cos (2t-2\varphi \,-\pi )-c\\ \,\,\,+\,|\cos (t-\varphi +v\,\cos (t-\varphi ))+\,m\,\cos (2t-2\varphi -\pi )-\,c|)],\end{array}$$

where *t* is time in radians, $b$ is baseline melatonin level ($b\, > 0$), $c$ is the peak width ($-1\,\le c\, < 1$), $H$ is peak melatonin level ($H\, > 0$), $m$ is a bimodality parameter ($0\,\le m\, < 1$), $\varphi$ is phase in radians ($0\le \varphi \le 2\pi$), and $v$ is a skewness parameter ($-5\,\le v\,\le 5$).

The fitted 24-h melatonin profile was selected, starting from the first increase above baseline melatonin levels. The 24-h profile was then extrapolated backwards onto the 7 days of data prior to the CR, thus covering the two baseline laboratory days and 5 days of at-home monitoring on a fixed sleep schedule. Melatonin data were scaled to within [0 1] to account for inter-individual variability in melatonin secretion levels^67^, and normally distributed random noise was added with standard deviation of 0.01.

*aMT6s:* The urinary aMT6s reference waveform was defined based on aMT6s secretion measured over 48 hours either (i) covering the two baseline days in the laboratory (FS phase); (ii) following a week of monitored HS; (iii) following a series of day/evening shifts or days off; or (iv) following a series of consecutive night shifts. aMT6s excretion rate was calculated by multiplying the concentration of aMT6s in each sample (ng/mL) by the volume of the sample and dividing by the duration of the collection interval^31^. Time points corresponding to excretion rates were defined as midpoints of the time intervals between samples. A cosine curve function was fitted to the data using nonlinear least squares, assuming a 24-h period, using the following formula:

$$f(t)=b+a\,\cos (\frac{2\pi }{24}(t-\varphi )),$$

where $t$ is time in minutes, $a$ is amplitude of the aMT6s rhythm ($0\, < a\, < 10000$), $b$ is the baseline rate of change ($0\, < b\, < 10000$), and $\varphi$ is the phase offset in minutes ($0\le \varphi  < 1440$), implemented with starting parameters of $a=300,\,\,c=500,\,\,\varnothing =24$. Data were then resampled to 1-minute timestamps, and the 24-h profile determined, starting at the cosine fit minimum.

For the non-shift work datasets, and the shift work datasets that used a participants’ first aMT6s assessment, the 24-h profile was extrapolated backwards to cover the period of ambulatory recording of light and skin temperature. For the shift work datasets that used participants’ second or third aMT6s assessment, a shifting aMT6s rhythm was generated between the two known acrophase times before and after. The period for the shifting waveform was calculated as 24 plus the daily shift in hours between the two acrophase times. For participants where there was no available aMT6s assessment on the first night shift, aMT6s acrophase from the day shift was substituted. This assumption was checked by visual inspection of sleep-wake patterns between day assessment and first night shift^25^. The fitted curves were then scaled to [0 1] to account for inter-individual variability in aMT6s secretion levels, and normally distributed random noise with standard deviation of 0.01 was added.

#### Circadian phase calculation

As reported by Kolodyazhniy, *et al*.^15^, circadian phase was calculated using the center of gravity (CoG) method:

$$CoG=\,\frac{{\sum }_{t=tstart}^{tstart+24}t(f(t)-\,b)}{{\sum }_{t=tstart}^{tstart+24}(f(t)-b)}$$

where $t$ is time in minutes, $b$ is the baseline found from fitting the BSBCF curve (melatonin) or cosine curve (aMT6s), and ${t}_{start}$ was time point of the minimum value.

#### Data alignment

Following all processing steps described above, data were averaged into 30-minute bins, with the timestamp in the middle of each bin^15^. Bins were timed to start either on the hour or half hour (e.g., 6:00 or 6:30 h). Data were cut to align with the shortest available input data stream – light or skin temperature – to ensure the same time-points were included as inputs to the model. For our implementation, we required each dataset to be a minimum length of 1 day to be included as an input for model training.

### Neural network model

The structure of the artificial neural network model replicates the network previously published by Kolodyazhniy, *et al*.^15^. The artificial neural network was a multilayer perceptron with 5 neurons, which each compute a weighted sum of its inputs plus a bias term^21^. For a schematic of the network structure see Fig. 2A.

We trained the model over nine different combinations of input variables (light, skin temperature, and activity) and four different sleep-wake dataset combinations. Primary results presented are all from the input combination of blue irradiance and skin temperature. Results from the other input combinations are summarized in Supplementary Table S2.

The neural network model was implemented using MATLAB R2016b with the Neural Network Toolbox (The MathWorks, Natick, MA, USA). First, we trained two neural network models with inputs collected on the FS schedule, implemented first using all 11 skin temperature sensors (combined into 6 variables), and then using only a single wrist-worn temperature sensor to determine the feasibility of using a single sensor, which could be easily implemented as a wearable device. The reference circadian rhythm was salivary melatonin measured during the constant routine.

We then trained two neural network models with the same inputs as described above, collected over both the FS and HS schedules, with reference circadian rhythms from urinary aMT6s. Models were then trained using all diurnal sleep schedules: datasets collected over the fixed and habitual sleep schedules on the non-shift work protocol, plus over the diurnal schedules on the shift work protocol, with reference circadian waveform of urinary aMT6s. Finally, two neural network models were trained on data from the shift work protocol (single temperature sensor only): one using inputs from the diurnal shift schedules, and another using inputs collected from the consecutive night shift schedules.

#### Model training and validation

Model training and validation steps replicate those published by Kolodyazhniy, *et al*.^15^. Prior to input into the model ambulatory data streams (light, skin temperature) were scaled to the group mean and standard deviation of the input data streams for n-1 participants. Time lags were added to the input variables: for light data, lags were added in the range 0–24 hours in 30-minute steps, resulting in a total of 49 light input variables. For skin temperature, lags were added in the range 0–5 hours in 30-minute steps, resulting in a total of 11 skin temperature input variables when using a single temperature variable, or 66 input variables when all 6 skin temperature variables were used. The number of weights varied depending on the model. There were a total of 586 weights for the models using blue irradiance and all skin temperature variables ((66 + 49 + 1) × 5 + (5 + 1) = 586), and 311 weights when using blue irradiance and a single skin temperature variable ((11 + 49 + 1) × 5 + (5 + 1) = 311). Only data collected outside the laboratory were used for model validation (i.e., data collected during in-laboratory baseline days in the non-shift work study were excluded) to simulate data collection in real-world conditions.

Model weights were found using resilient backpropagation with the built-in MATLAB function ‘trainrp’. For each model, the neural network training was repeated 100 times, each time using different random initialization of the network weights. We trialed training for up to 200 or 300 times, with minimal impact on model performance. The models were trained using leave-one-out cross validation to ensure participant-independent validation, whereby the model was trained on data from n-1 participants, with performance then validated using the left out participant. A final model was trained using data from all participants (n), for application to independent datasets.

#### Predictions using final trained networks

In addition to training models using leave-one-out cross-validation, two final trained network models were used to generate predictions on independent shift work datasets. First, predictions were obtained by inputting ambulatory data collected in the shift work study (both SWday and SWnight datasets) using the final trained non-shift work model. Second, predictions were obtained by inputting the ambulatory data over the night shift work schedule (SWnight) into the final trained diurnal model.

#### Calculation of predicted phase

Predicted circadian phase was determined by fitting a curve to the predicted rhythm generated by the neural network (BSBCF curve for networks trained on salivary melatonin; cosine curve for networks trained on urinary aMT6s), and circadian phase found using the center of gravity method described above. Predicted phase for each participant was calculated using a model trained on the data, excluding that participant.

### Comparative methods of phase estimation

Two alternative approaches were used for prediction of circadian phase. First, given the use of sleep-wake timing to estimate circadian phase, including for diagnosis of circadian rhythm disorders^9,68^, average mid-sleep timing over each dataset was tested as a proxy for aMT6s acrophase^69,70^. Second, to assess model performance relative to chance, circadian phase was estimated on the diurnal and SWnight datasets by randomly assigning time values either (a) within a defined range of urinary aMT6s acrophase observed in sighted, normally entrained individuals^27^; or (b) from a surrogate dataset based on random sampling of measured urinary aMT6s acrophase times within each group of datasets.

### Data analysis

Prediction error was calculated for each model by subtracting the predicted circadian phase from experimental circadian phase on the same date. For each model, the following summary statistics were calculated based on prediction error: mean, median, standard deviation, mean absolute difference, root mean square error, and the percentage of predictions within ±15, ±30, ±60 and ±120 minutes of measured phase time. Correlations between measured and predicted circadian phase, and between predicted phase across datasets, were calculated using circular statistics^71^, as linear statistics may not be appropriate due to the wide range in circadian phase^72^.

To compare the relationship between melatonin and aMT6s phase, paired-samples t-tests were used to examine the relationship between the timing of predicted melatonin and aMT6s phase, and between measured melatonin and aMT6s phase on the FS schedule. Paired-samples t-tests were also used to compare predictions using all 11 vs. a single skin temperature sensor for FS and HS + FS datasets. Pearson’s correlations were used to examine the relationship between model predictions using all 11 vs. a single skin temperature sensor. Independent-samples t-tests were used to compare prediction error between sleep schedules. Phase angle of entrainment was calculated for each dataset as the difference between urinary aMT6s acrophase and average sleep onset time for an individual. Relationships between prediction error and participant characteristics including age, MEQ score, BMI, measured aMT6s phase shift, measured reference phase, phase angle of entrainment, and length of the dataset were examined for each model using Pearson’s correlations.

## Supplementary information

Supplementary Information

## Data Availability

Materials and data in this publication can be requested via the CRC for Alertness, Safety and Productivity (Alertness CRC) by emailing inquiries@alertnesscrc.com.
