# Supplementary material for: Generalizability of A Neural Network Model for Circadian Phase Prediction in Real-World Conditions
Source: Sci Rep. 2019 Jul 29;9:11001. doi: 10.1038/s41598-019-47311-4 (PMC6662750; doi:10.1038/s41598-019-47311-4)
Supplement: Supplementary file 1 — Supplementary Information [file 41598_2019_47311_MOESM1_ESM.docx]

## Supplementary information

**Generalizability of a neural network model for circadian phase prediction in real-world conditions**

Julia E. Stone^1,2^, Andrew J. K. Phillips^1,2^, Suzanne Ftouni^1,2^, Michelle Magee^1,2^, Mark Howard^1,2,5^, Steven W. Lockley^1,2,3,4^, Tracey L. Sletten^1,2^, Clare Anderson^1,2^, Shantha M. W. Rajaratnam^1,2^, Svetlana Postnova^1,6^

*^1^Cooperative Research Centre for Alertness, Safety and Productivity, Melbourne, Victoria, Australia*

*^2^ School of Psychological Sciences, Monash University, Melbourne, Victoria, Australia*

*^3^Division of Sleep and Circadian Disorders, Departments of Medicine and Neurology, Brigham and Women’s Hospital, Boston, Massachusetts, USA*

*^4^Division of Sleep Medicine, Harvard Medical School, Boston, Massachusetts, USA*

*^5^Institute for Breathing and Sleep, Austin Health, Victoria, Australia*

*^6^ School of Physics, University of Sydney, Sydney, New South Wales, Australia*

**Table S1.** Summary of available raw data (%)

|  | **Fixed Sleep** | | | | | **Habitual Sleep** | | | | |
| --- | --- | --- | --- | --- | --- | --- | --- | --- | --- | --- |
|  | n | Mean | SD | Min | Max | n | Mean | SD | Min | Max |
| *Light* |  |  |  |  |  |  |  |  |  |  |
| Pre-cleaning | 15 | 99.88 | 0.22 | 99.17 | 100.00 | 14 | 99.64 | 0.81 | 97.30 | 100.00 |
| Cleaned | 15 | 86.41 | 11.80 | 59.12 | 98.06 | 14 | 88.88 | 4.76 | 76.64 | 95.11 |
| *Skin Temperature (wrist sensor)* | | |  |  |  |  |  |  |  |  |
| Pre-cleaning Step 1 | 15 | 100.00 | 0.01 | 99.95 | 100.00 | 14 | 99.99 | 0.02 | 99.93 | 100.00 |
| Pre-cleaning Step 2 | 15 | 93.17 | 6.65 | 79.88 | 100.00 | 14 | 89.21 | 14.35 | 59.52 | 100.00 |
| Cleaned | 15 | 92.46 | 6.39 | 79.55 | 99.65 | 14 | 88.18 | 13.89 | 59.50 | 99.98 |
|  |  |  |  |  |  |  |  |  |  |  |
|  | **Shift Work Diurnal** | | | | | **Shift Work Nights** | | | | |
|  | n | Mean | SD | Min | Max | n | Mean | SD | Min | Max |
| *Light* |  |  |  |  |  |  |  |  |  |  |
| Pre-cleaning | 29 | 94.14 | 6.70 | 74.61 | 99.77 | 19 | 93.46 | 6.90 | 72.57 | 99.85 |
| Cleaned | 29 | 85.35 | 7.50 | 70.19 | 95.83 | 19 | 84.35 | 6.76 | 67.02 | 94.43 |
| *Skin Temperature (wrist sensor)* | | |  |  |  |  |  |  |  |  |
| Pre-cleaning Step 1 | 29 | 93.99 | 11.51 | 63.61 | 100.00 | 19 | 93.33 | 11.89 | 66.34 | 100.00 |
| Pre-cleaning Step 2 | 29 | 87.22 | 12.86 | 61.07 | 99.74 | 19 | 88.67 | 11.60 | 65.64 | 99.81 |
| Cleaned | 29 | 86.08 | 12.62 | 60.90 | 99.02 | 19 | 87.18 | 11.81 | 65.40 | 99.12 |

Note: for light data pre-cleaning involved removal of artefact data due to sleeve covering (i.e. data <1 lux during wake times were removed). For skin temperature data, pre-cleaning step 1 involved removal of skin temperature data based on participant report (Fixed Sleep and Habitual Sleep) or where the actigraph was marked as off-wrist (Shift Work Diurnal and Shift Work Nights). Pre-cleaning step 2 involved removal of all data < 20 degrees Celsius and outliers >3 SD from the mean.

**Table S2.** Summary of models trained by input variable and sleep schedule

|  |  |  | **Sleep Schedule** | | | | |
| --- | --- | --- | --- | --- | --- | --- | --- |
|  |  |  | **Fixed Sleep** | **Fixed + Habitual Sleep** | **Diurnal Sleep** | **Shift Work Day Shifts** | **Shift Work Night Shifts** |
| **Inputs** | **One variable** | Blue irradiance | ✔ * | ✔ | ✔ | ✔ | ✔ |
|  |  | White lux | ✔ * | ✔ | ✔ | ✔ | ✔ |
|  |  | Skin temperature (all) | ✔ * | ✔ | N/A | N/A | N/A |
|  |  | Skin temperature (single) | ✔ * | ✔ | ✔ | ✔ | ✔ |
|  |  | Activity | ✔ * | ✔ | ✔ | ✔ | ✔ |
|  | **Two variables** | Blue irradiance + SkinT (all) | ✔ * | ✔ | N/A | N/A | N/A |
|  |  | Blue irradiance + SkinT (single) | ✔ * | ✔ | ✔ | ✔ | ✔ |
|  |  | Blue irradiance + Activity | ✔ * | ✔ | ✔ | ✔ | ✔ |
|  |  | White lux + Skin temperature (all) | ✔ * | ✔ | N/A | N/A | N/A |
|  |  | White lux + Skin temperature (single) | ✔ * | ✔ | ✔ | ✔ | ✔ |
|  |  | Activity + Skin temperature (all) | ✔ * | ✔ | N/A | N/A | N/A |
|  |  | Activity + Skin temperature (single) | ✔ * | ✔ | ✔ | ✔ | ✔ |
|  | **Three variables** | Blue irradiance + Activity + Skin temperature (all) | ✔ * | ✔ | N/A | N/A | N/A |
|  |  | Blue irradiance + Activity + Skin temperature (single) | ✔ * | ✔ | ✔ | ✔ | ✔ |

Note: * trained on salivary melatonin and aMT6s. All networks trained on aMT6s. Skin temperature (all) had inputs from sensors on distal and proximal skin sites (shoulders, sternum, wrists, thighs, calves, feet). Skin temperature (single) had inputs from the non-dominant wrist only.

|  |  |  | |  | |  | | **Prediction error absolute values (minutes)** | | | | | | | | | **Prediction error (minutes)** | | | | | | | |  | |  | |  | | **Percentage predicted within +/-** | | | | | | | |  |  |  |
| --- | --- | --- | --- | --- | --- | --- | --- | --- | --- | --- | --- | --- | --- | --- | --- | --- | --- | --- | --- | --- | --- | --- | --- | --- | --- | --- | --- | --- | --- | --- | --- | --- | --- | --- | --- | --- | --- | --- | --- | --- | --- |
| **Sleep Schedule** | **marker** | **Inputs** | | **n** | | **temp** | | **Mean abs error** | | **Median abs error** | | **SD abs error** | **Range abs error** | **Min abs error** | **Max abs error** | | **Mean error** | | **Median error** | | **SD error** | | **Range error** | | **RMSE** | | **r** | | **p** | | **15 mins** | | **30 mins** | | **60 mins** | | **120 mins** | | |  |  |
| **Fixed sleep (non-shift work study)** | | | | |  | |  | |  | |  |  |  |  | |  | |  | |  | |  | |  | |  | |  | |  | |  | |  | |  | |  | | |  |
|  | melatonin | Blue irradiance + skin temperature + activity | | 15 | | single | | 36.94 | | 25.89 | | 31.44 | 97.52 | 4.08 | 101.60 | | 5.47 | | 6.05 | | 49.18 | | 192.46 | | 47.83 | | 0.46 | | 0.08 | | 33.33 | | 53.33 | | 80.00 | | 100.00 | | |  |  |
|  | melatonin | Blue irradiance + activity | | 15 | | single | | 37.42 | | 39.49 | | 32.82 | 111.80 | 0.01 | 111.81 | | -1.89 | | 5.50 | | 50.73 | | 190.02 | | 49.05 | | 0.47 | | 0.08 | | 33.33 | | 46.67 | | 73.33 | | 100.00 | | |  |  |
|  | melatonin | Skin temperature + activity | | 15 | | single | | 38.98 | | 29.98 | | 36.95 | 127.15 | 5.06 | 132.21 | | 3.02 | | 9.40 | | 54.62 | | 241.01 | | 52.85 | | 0.44 | | 0.10 | | 26.67 | | 53.33 | | 80.00 | | 93.33 | | |  |  |
|  | melatonin | White lux + skin temperature | | 15 | | single | | 39.58 | | 29.04 | | 31.31 | 98.37 | 5.58 | 103.95 | | 6.82 | | 16.76 | | 51.08 | | 192.23 | | 49.81 | | 0.44 | | 0.10 | | 20.00 | | 60.00 | | 73.33 | | 100.00 | | |  |  |
|  | melatonin | Blue irradiance + skin temperature | | 15 | | single | | 40.86 | | 27.01 | | 31.97 | 99.23 | 7.06 | 106.29 | | 1.33 | | 11.48 | | 53.00 | | 194.61 | | 51.23 | | 0.41 | | 0.12 | | 26.67 | | 53.33 | | 66.67 | | 100.00 | | |  |  |
|  | melatonin | White lux | | 15 | | single | | 41.62 | | 34.62 | | 33.25 | 108.41 | 4.11 | 112.51 | | 0.72 | | 4.11 | | 54.41 | | 210.70 | | 52.57 | | 0.36 | | 0.18 | | 13.33 | | 46.67 | | 73.33 | | 100.00 | | |  |  |
|  | melatonin | Blue irradiance | | 15 | | single | | 41.70 | | 30.75 | | 34.53 | 102.86 | 3.50 | 106.36 | | 1.05 | | 12.01 | | 55.27 | | 200.84 | | 53.40 | | 0.39 | | 0.15 | | 26.67 | | 46.67 | | 66.67 | | 100.00 | | |  |  |
|  | melatonin | Activity | | 15 | | single | | 46.85 | | 36.20 | | 41.49 | 134.66 | 1.67 | 136.33 | | 2.20 | | 3.38 | | 63.78 | | 257.91 | | 61.65 | | 0.36 | | 0.19 | | 20.00 | | 33.33 | | 73.33 | | 86.67 | | |  |  |
|  | melatonin | Skin temperature | | 15 | | single | | 61.75 | | 55.76 | | 34.15 | 116.84 | 16.42 | 133.25 | | 48.88 | | 54.98 | | 51.88 | | 196.31 | | 70.02 | | 0.45 | | 0.09 | | 0.00 | | 13.33 | | 53.33 | | 86.67 | | |  |  |
|  |  |  | |  | |  | |  | |  | |  |  |  |  | |  | |  | |  | |  | |  | |  | |  | |  | |  | |  | |  | | |  |  |
|  | melatonin | Blue irradiance + skin temperature + activity | | 15 | | all | | 37.98 | | 40.79 | | 26.06 | 81.61 | 3.19 | 84.80 | | 2.94 | | 8.24 | | 47.06 | | 156.68 | | 45.56 | | 0.53 | | 0.04 | | 26.67 | | 40.00 | | 80.00 | | 100.00 | | |  |  |
|  | melatonin | Skin temperature + activity | | 15 | | all | | 38.42 | | 24.99 | | 33.60 | 116.84 | 6.34 | 123.18 | | 4.68 | | 14.36 | | 51.83 | | 206.66 | | 50.29 | | 0.46 | | 0.08 | | 26.67 | | 60.00 | | 80.00 | | 93.33 | | |  |  |
|  | melatonin | White lux + skin temperature | | 15 | | all | | 40.24 | | 30.19 | | 32.12 | 86.84 | 1.51 | 88.35 | | 3.01 | | 13.41 | | 52.51 | | 176.00 | | 50.82 | | 0.36 | | 0.19 | | 26.67 | | 46.67 | | 66.67 | | 100.00 | | |  |  |
|  | melatonin | Blue irradiance + skin temperature | | 15 | | all | | 42.49 | | 39.43 | | 25.24 | 70.69 | 14.21 | 84.91 | | 5.67 | | 17.82 | | 50.36 | | 166.03 | | 48.98 | | 0.43 | | 0.11 | | 6.67 | | 40.00 | | 73.33 | | 100.00 | | |  |  |
|  | melatonin | Skin temperature | | 15 | | all | | 60.42 | | 54.81 | | 34.31 | 105.73 | 17.24 | 122.97 | | 32.05 | | 40.80 | | 63.15 | | 237.91 | | 68.92 | | 0.14 | | 0.61 | | 0.00 | | 20.00 | | 66.67 | | 86.67 | | |  |  |
|  | | | | |  | |  | |  | | | | | | | | |  | | | | | | | |  | |  | |  | |  | | | | | | | | |  |
|  | | | | |  | |  | |  | | | | | | | | |  | | | | | | | |  | |  | |  | |  | | | | | | | | |  |
|  | | | | |  | |  | | **Prediction error absolute values (minutes)** | | | | | | | | | **Prediction error (minutes)** | | | | | | | |  | |  | |  | | **Percentage predicted within +/-** | | | | | | | | |  |
| **Sleep Schedule** | **marker** | **Inputs** | | **n** | | **temp** | | **Mean abs error** | | **Median abs error** | | **SD abs error** | **Range abs error** | **Min abs error** | **Max abs error** | | **Mean error** | | **Median error** | | **SD error** | | **Range error** | | **RMSE** | | **r** | | **p** | | **15 mins** | | **30 mins** | | **60 mins** | | **120 mins** | | |  |  |
| **Fixed and habitual sleep (non-shift work study)** | | | | | |  | |  | |  | |  |  |  |  | |  | |  | |  | |  | |  | |  | |  | |  | |  | |  | |  | | |  |  |
|  | aMT6s | Blue irradiance + skin temperature | | 27 | | single | | 50.85 | | 40.98 | | 42.22 | 149.86 | 2.74 | 152.60 | | -0.91 | | -4.16 | | 66.83 | | 277.90 | | 65.59 | | 0.45 | | 0.02 | | 22.22 | | 37.04 | | 66.67 | | 88.89 | | |  |  |
|  | aMT6s | Blue irradiance + skin temperature + activity | | 27 | | single | | 52.08 | | 43.98 | | 43.83 | 156.43 | 0.20 | 156.63 | | 2.33 | | -6.15 | | 68.79 | | 268.42 | | 67.55 | | 0.43 | | 0.02 | | 25.93 | | 40.74 | | 66.67 | | 92.59 | | |  |  |
|  | aMT6s | Blue irradiance | | 27 | | single | | 53.35 | | 42.13 | | 44.80 | 157.06 | 4.91 | 161.97 | | -4.61 | | -7.02 | | 70.29 | | 285.27 | | 69.13 | | 0.39 | | 0.04 | | 22.22 | | 40.74 | | 66.67 | | 85.19 | | |  |  |
|  | aMT6s | White lux + skin temperature | | 27 | | single | | 53.87 | | 50.21 | | 41.82 | 152.38 | 1.37 | 153.75 | | -0.22 | | -8.46 | | 69.01 | | 256.10 | | 67.72 | | 0.43 | | 0.03 | | 14.81 | | 44.44 | | 62.96 | | 92.59 | | |  |  |
|  | aMT6s | White lux | | 27 | | single | | 56.63 | | 46.91 | | 41.88 | 143.91 | 2.69 | 146.60 | | -1.15 | | -11.15 | | 71.29 | | 261.70 | | 69.97 | | 0.37 | | 0.06 | | 14.81 | | 33.33 | | 62.96 | | 88.89 | | |  |  |
|  | aMT6s | Blue irradiance + activity | | 27 | | single | | 57.01 | | 43.09 | | 42.29 | 147.98 | 2.84 | 150.82 | | 0.28 | | -2.84 | | 71.85 | | 275.19 | | 70.51 | | 0.38 | | 0.05 | | 18.52 | | 25.93 | | 66.67 | | 85.19 | | |  |  |
|  | aMT6s | Skin temperature + activity | | 27 | | single | | 59.68 | | 46.66 | | 47.07 | 182.41 | 0.56 | 182.98 | | 2.72 | | -1.51 | | 76.85 | | 309.34 | | 75.47 | | 0.36 | | 0.06 | | 14.81 | | 29.63 | | 62.96 | | 81.48 | | |  |  |
|  | aMT6s | Skin temperature | | 27 | | single | | 62.39 | | 56.73 | | 50.55 | 216.75 | 1.08 | 217.84 | | 36.44 | | 29.00 | | 72.24 | | 292.49 | | 79.71 | | 0.47 | | 0.01 | | 11.11 | | 33.33 | | 51.85 | | 88.89 | | |  |  |
|  | aMT6s | Activity | | 27 | | single | | 65.06 | | 53.90 | | 46.67 | 178.30 | 2.87 | 181.17 | | -3.82 | | -18.03 | | 80.99 | | 300.68 | | 79.56 | | 0.30 | | 0.13 | | 7.41 | | 29.63 | | 55.56 | | 92.59 | | |  |  |
|  |  |  | |  | |  | |  | |  | |  |  |  |  | |  | |  | |  | |  | |  | |  | |  | |  | |  | |  | |  | | |  |  |
|  | aMT6s | Blue irradiance + skin temperature | | 26 | | all | | 48.71 | | 37.76 | | 44.19 | 150.50 | 0.81 | 151.32 | | 1.88 | | -1.46 | | 66.46 | | 256.29 | | 65.20 | | 0.46 | | 0.02 | | 26.92 | | 42.31 | | 65.38 | | 92.31 | | |  |  |
|  | aMT6s | Blue irradiance + skin temperature + activity | | 26 | | all | | 52.17 | | 37.79 | | 47.84 | 157.50 | 2.03 | 159.53 | | 2.35 | | -5.96 | | 71.51 | | 271.79 | | 70.16 | | 0.38 | | 0.06 | | 30.77 | | 42.31 | | 57.69 | | 88.46 | | |  |  |
|  | aMT6s | White lux + skin temperature | | 26 | | all | | 53.96 | | 52.45 | | 41.47 | 153.52 | 0.27 | 153.79 | | 0.68 | | -6.43 | | 68.90 | | 260.68 | | 67.57 | | 0.41 | | 0.04 | | 23.08 | | 34.62 | | 57.69 | | 92.31 | | |  |  |
|  | aMT6s | Skin temperature + activity | | 26 | | all | | 55.92 | | 44.07 | | 49.81 | 183.84 | 0.46 | 184.31 | | 6.76 | | -2.59 | | 75.40 | | 292.54 | | 74.25 | | 0.40 | | 0.04 | | 30.77 | | 42.31 | | 57.69 | | 88.46 | | |  |  |
|  | aMT6s | Skin temperature | | 26 | | all | | 61.17 | | 48.99 | | 50.27 | 193.01 | 0.50 | 193.51 | | 31.75 | | 20.36 | | 73.28 | | 281.78 | | 78.56 | | 0.44 | | 0.02 | | 11.54 | | 34.62 | | 57.69 | | 80.77 | | |  |  |
|  | | | | | | | | |  | |  |  |  |  | |  | |  | |  | |  | |  | |  | |  | |  | |  | |  | |  | |  | | | |
|  | | | | | | | | |  | |  |  |  |  | |  | |  | |  | |  | |  | |  | |  | |  | |  | |  | |  | |  | | | |
|  | | | | | | | | |  | |  |  |  |  | |  | |  | |  | |  | |  | |  | |  | |  | |  | |  | |  | |  | | | |
|  | | | | | | | | |  | |  |  |  |  | |  | |  | |  | |  | |  | |  | |  | |  | |  | |  | |  | |  | | | |
|  | | | | | | | | |  | |  |  |  |  | |  | |  | |  | |  | |  | |  | |  | |  | |  | |  | |  | |  | | | |
|  | | | | | | | | |  | |  |  |  |  | |  | |  | |  | |  | |  | |  | |  | |  | |  | |  | |  | |  | | | |
|  | | | | | | | | |  | |  |  |  |  | |  | |  | |  | |  | |  | |  | |  | |  | |  | |  | |  | |  | | | |
|  | | | | | | | | | **Prediction error absolute values (minutes)** | | | | | | | | | **Prediction error (minutes)** | | | | | | | |  | |  | |  | | **Percentage predicted within +/-** | | | | | | | | | |
| **Sleep Schedule** | **marker** | **Inputs** | | **n** | | **temp** | | **Mean abs error** | | **Median abs error** | | **SD abs error** | **Range abs error** | **Min abs error** | **Max abs error** | | **Mean error** | | **Median error** | | **SD error** | | **Range error** | | **RMSE** | | **r** | | **p** | | **15 mins** | | **30 mins** | | **60 mins** | | **120 mins** | | |  |  |
| **All Diurnal (fixed and habitual sleep plus day schedule shift workers)** | | | | | | | | | | | |  |  |  |  | |  | |  | |  | |  | |  | |  | |  | |  | |  | |  | |  | | |  |  |
|  | aMT6s | Blue irradiance | | 56 | | single | | 59.29 | | 43.76 | | 63.21 | 334.51 | 0.48 | 334.99 | | 6.25 | | -3.07 | | 86.80 | | 538.17 | | 86.25 | | -0.13 | | 0.36 | | 25.00 | | 39.29 | | 66.07 | | 87.50 | | |  |  |
|  | aMT6s | Blue irradiance + skin temperature | | 56 | | single | | 62.84 | | 48.60 | | 67.16 | 312.24 | 0.42 | 312.66 | | -0.95 | | -3.27 | | 92.36 | | 604.60 | | 91.54 | | -0.11 | | 0.44 | | 19.64 | | 41.07 | | 66.07 | | 87.50 | | |  |  |
|  | aMT6s | Blue irradiance + activity | | 56 | | single | | 65.17 | | 39.16 | | 71.95 | 367.29 | 4.70 | 371.99 | | 9.91 | | -2.74 | | 96.97 | | 564.73 | | 96.60 | | -0.05 | | 0.73 | | 16.07 | | 35.71 | | 64.29 | | 85.71 | | |  |  |
|  | aMT6s | White lux | | 56 | | single | | 66.30 | | 37.28 | | 95.36 | 565.13 | 1.17 | 566.30 | | 16.15 | | -2.25 | | 115.34 | | 766.68 | | 115.44 | | -0.02 | | 0.89 | | 25.00 | | 42.86 | | 64.29 | | 87.50 | | |  |  |
|  | aMT6s | White lux + skin temperature | | 56 | | single | | 67.16 | | 37.12 | | 104.28 | 637.52 | 1.96 | 639.47 | | 18.03 | | -0.92 | | 123.03 | | 833.63 | | 123.25 | | 0.02 | | 0.88 | | 23.21 | | 46.43 | | 64.29 | | 89.29 | | |  |  |
|  | aMT6s | Activity | | 56 | | single | | 69.96 | | 40.69 | | 90.65 | 580.05 | 0.35 | 580.40 | | 11.87 | | -7.06 | | 114.27 | | 731.86 | | 113.87 | | -0.06 | | 0.64 | | 16.07 | | 32.14 | | 60.71 | | 85.71 | | |  |  |
|  | aMT6s | Blue irradiance + skin temperature + activity | | 56 | | single | | 70.27 | | 44.61 | | 94.18 | 617.04 | 0.19 | 617.23 | | 12.40 | | -6.43 | | 117.22 | | 832.57 | | 116.83 | | -0.10 | | 0.48 | | 16.07 | | 35.71 | | 58.93 | | 87.50 | | |  |  |
|  | aMT6s | Skin temperature + activity | | 56 | | single | | 72.71 | | 45.85 | | 91.18 | 607.99 | 0.12 | 608.11 | | 11.67 | | -7.98 | | 116.43 | | 764.42 | | 115.98 | | -0.10 | | 0.45 | | 14.29 | | 30.36 | | 62.50 | | 91.07 | | |  |  |
|  | aMT6s | Skin temperature | | 56 | | single | | 78.69 | | 60.76 | | 91.30 | 580.31 | 0.16 | 580.47 | | 28.81 | | 32.99 | | 117.45 | | 908.17 | | 119.91 | | -0.10 | | 0.48 | | 17.86 | | 25.00 | | 48.21 | | 82.14 | | |  |  |
| **Day schedule shift workers** | | | | |  | |  | |  | |  |  |  |  | |  | |  | |  | |  | |  | |  | |  | |  | |  | |  | |  | |  | | |  |
|  | aMT6s | Blue irradiance + skin temperature | | 29 | | single | | 65.68 | | 38.28 | | 80.34 | 324.44 | 3.34 | 327.79 | | 9.36 | | -9.21 | | 104.08 | | 541.49 | | 102.69 | | -0.31 | | 0.10 | | 17.24 | | 41.38 | | 68.97 | | 86.21 | | |  |  |
|  | aMT6s | Blue irradiance | | 29 | | single | | 68.73 | | 41.32 | | 81.62 | 346.69 | 4.19 | 350.88 | | 14.92 | | 7.62 | | 106.41 | | 567.04 | | 105.62 | | -0.26 | | 0.17 | | 20.69 | | 37.93 | | 65.52 | | 86.21 | | |  |  |
|  | aMT6s | Blue irradiance + activity | | 29 | | single | | 75.54 | | 38.65 | | 89.57 | 348.52 | 1.48 | 350.00 | | 21.09 | | 8.99 | | 116.07 | | 532.88 | | 115.99 | | -0.23 | | 0.23 | | 10.34 | | 34.48 | | 68.97 | | 82.76 | | |  |  |
|  | aMT6s | White lux + skin temperature | | 29 | | single | | 75.92 | | 31.88 | | 107.19 | 440.66 | 1.61 | 442.26 | | 26.53 | | -1.61 | | 129.34 | | 620.12 | | 129.84 | | -0.14 | | 0.47 | | 31.03 | | 48.28 | | 62.07 | | 86.21 | | |  |  |
|  | aMT6s | Blue irradiance + skin temperature + activity | | 29 | | single | | 78.98 | | 36.01 | | 102.96 | 463.63 | 0.51 | 464.14 | | 20.18 | | -2.76 | | 128.99 | | 647.14 | | 128.35 | | -0.25 | | 0.18 | | 13.79 | | 37.93 | | 58.62 | | 82.76 | | |  |  |
|  | aMT6s | White lux | | 29 | | single | | 82.11 | | 31.27 | | 134.32 | 600.68 | 0.41 | 601.09 | | 32.28 | | 0.78 | | 154.75 | | 807.28 | | 155.44 | | -0.06 | | 0.76 | | 34.48 | | 48.28 | | 62.07 | | 86.21 | | |  |  |
|  | aMT6s | Skin temperature + activity | | 29 | | single | | 83.85 | | 47.07 | | 123.62 | 619.23 | 0.40 | 619.63 | | 25.96 | | -9.64 | | 147.87 | | 785.21 | | 147.60 | | -0.16 | | 0.40 | | 17.24 | | 31.03 | | 62.07 | | 79.31 | | |  |  |
|  | aMT6s | Activity | | 29 | | single | | 83.87 | | 46.22 | | 117.77 | 575.99 | 3.37 | 579.36 | | 24.10 | | -18.38 | | 143.36 | | 747.45 | | 142.91 | | -0.15 | | 0.43 | | 13.79 | | 31.03 | | 62.07 | | 82.76 | | |  |  |
|  | aMT6s | Skin temperature | | 29 | | single | | 94.73 | | 58.97 | | 115.69 | 575.13 | 4.06 | 579.19 | | 23.83 | | 48.13 | | 148.63 | | 910.27 | | 147.97 | | -0.19 | | 0.33 | | 20.69 | | 24.14 | | 51.72 | | 75.86 | | |  |  |
|  | | | | |  | |  | |  | |  |  |  |  | |  | |  | |  | |  | |  | |  | |  | |  | |  | |  | |  | |  | | |  |
|  | | | | |  | |  | |  | |  |  |  |  | |  | |  | |  | |  | |  | |  | |  | |  | |  | |  | |  | |  | | |  |
|  | | | | |  | |  | |  | |  |  |  |  | |  | |  | |  | |  | |  | |  | |  | |  | |  | |  | |  | |  | | |  |
|  | | | | |  | |  | | **Prediction error absolute values (minutes)** | | | | | | | | | **Prediction error (minutes)** | | | | | | | |  | |  | |  | | **Percentage predicted within +/-** | | | | | | | | |  |
| **Sleep Schedule** | **marker** | **Inputs** | | **n** | | **temp** | | **Mean abs error** | | **Median abs error** | | **SD abs error** | **Range abs error** | **Min abs error** | **Max abs error** | | **Mean error** | | **Median error** | | **SD error** | | **Range error** | | **RMSE** | | **r** | | **p** | | **15 mins** | | **30 mins** | | **60 mins** | | **120 mins** | | |  |  |
| **Night schedule shift workers** | | | | | |  | |  | |  | |  |  |  |  | |  | |  | |  | |  | |  | |  | |  | |  | |  | |  | |  | | |  |  |
|  | aMT6s | Blue irradiance + skin temperature | | 19 | | single | | 143.29 | | 115.28 | | 155.42 | 603.98 | 1.50 | 605.48 | | -22.11 | | -10.24 | | 212.86 | | 1013.81 | | 208.36 | | 0.10 | | 0.70 | | 21.05 | | 31.58 | | 42.11 | | 52.63 | | |  |  |
|  | aMT6s | Skin temperature + activity | | 19 | | single | | 154.08 | | 104.17 | | 166.83 | 592.73 | 8.46 | 601.20 | | -36.65 | | -11.65 | | 226.88 | | 1138.23 | | 223.85 | | 0.42 | | 0.07 | | 10.53 | | 10.53 | | 21.05 | | 68.42 | | |  |  |
|  | aMT6s | White lux | | 19 | | single | | 155.11 | | 108.56 | | 150.03 | 634.26 | 8.56 | 642.83 | | -42.42 | | -40.15 | | 214.48 | | 879.27 | | 213.03 | | -0.20 | | 0.41 | | 10.53 | | 15.79 | | 31.58 | | 52.63 | | |  |  |
|  | aMT6s | Blue irradiance | | 19 | | single | | 158.68 | | 131.76 | | 151.89 | 598.07 | 1.51 | 599.58 | | -53.02 | | -8.61 | | 216.05 | | 876.39 | | 216.87 | | -0.16 | | 0.52 | | 15.79 | | 21.05 | | 31.58 | | 47.37 | | |  |  |
|  | aMT6s | White lux + skin temperature | | 19 | | single | | 158.77 | | 133.23 | | 147.09 | 633.25 | 2.34 | 635.59 | | -33.98 | | -50.71 | | 216.85 | | 950.21 | | 213.79 | | -0.08 | | 0.73 | | 10.53 | | 10.53 | | 26.32 | | 47.37 | | |  |  |
|  | aMT6s | Skin temperature | | 19 | | single | | 163.99 | | 100.87 | | 170.94 | 655.58 | 6.64 | 662.22 | | -75.84 | | -62.85 | | 227.01 | | 1140.45 | | 233.61 | | 0.36 | | 0.13 | | 10.53 | | 10.53 | | 21.05 | | 63.16 | | |  |  |
|  | aMT6s | Blue irradiance + activity | | 19 | | single | | 173.14 | | 108.28 | | 189.77 | 673.04 | 10.27 | 683.31 | | -0.72 | | 10.27 | | 260.11 | | 1287.93 | | 253.17 | | -0.08 | | 0.74 | | 10.53 | | 15.79 | | 21.05 | | 63.16 | | |  |  |
|  | aMT6s | Activity | | 19 | | single | | 175.73 | | 112.55 | | 169.23 | 566.51 | 30.72 | 597.23 | | -57.95 | | -39.99 | | 240.19 | | 1059.33 | | 240.86 | | -0.01 | | 0.95 | | 0.00 | | 0.00 | | 21.05 | | 63.16 | | |  |  |
|  | aMT6s | Blue irradiance + skin temperature + activity | | 19 | | single | | 181.70 | | 112.14 | | 183.34 | 636.46 | 9.60 | 646.06 | | -70.72 | | -90.25 | | 251.36 | | 1074.34 | | 254.68 | | -0.03 | | 0.92 | | 5.26 | | 10.53 | | 15.79 | | 52.63 | | |  |  |
| **All datasets** | | |  | |  | |  | |  | |  |  |  |  | |  | |  | |  | |  | |  | |  | |  | |  | |  | |  | |  | |  | | |  |
|  | aMT6s | Blue irradiance + activity | | 77 | | single | | 122.94 | | 66.74 | | 132.82 | 543.38 | 4.39 | 547.78 | | 46.63 | | 15.26 | | 175.36 | | 928.14 | | 180.35 | | 0.21 | | 0.06 | | 7.79 | | 27.27 | | 45.45 | | 67.53 | | |  |  |
|  | aMT6s | Blue irradiance + skin temperature + activity | | 77 | | single | | 127.38 | | 60.86 | | 151.27 | 620.88 | 1.61 | 622.49 | | 39.99 | | 3.31 | | 194.17 | | 1144.66 | | 197.00 | | 0.18 | | 0.11 | | 14.29 | | 23.38 | | 48.05 | | 68.83 | | |  |  |
|  | aMT6s | White lux + skin temperature | | 77 | | single | | 128.57 | | 69.09 | | 157.92 | 699.03 | 0.30 | 699.34 | | 53.84 | | 7.33 | | 196.85 | | 1323.83 | | 202.84 | | 0.26 | | 0.02 | | 16.88 | | 29.87 | | 45.45 | | 68.83 | | |  |  |
|  | aMT6s | Blue irradiance | | 77 | | single | | 135.13 | | 68.37 | | 167.22 | 689.55 | 0.59 | 690.15 | | 59.94 | | 11.26 | | 206.93 | | 1246.33 | | 214.14 | | 0.19 | | 0.09 | | 15.58 | | 28.57 | | 42.86 | | 67.53 | | |  |  |
|  | aMT6s | Blue irradiance + skin temperature | | 77 | | single | | 135.25 | | 63.14 | | 174.85 | 706.32 | 3.16 | 709.48 | | 59.27 | | 7.81 | | 213.42 | | 1175.06 | | 220.16 | | 0.17 | | 0.14 | | 14.29 | | 29.87 | | 49.35 | | 68.83 | | |  |  |
|  | aMT6s | White lux | | 77 | | single | | 140.28 | | 79.84 | | 169.86 | 717.83 | 0.53 | 718.36 | | 64.54 | | 6.31 | | 211.11 | | 1324.84 | | 219.44 | | 0.20 | | 0.08 | | 20.78 | | 32.47 | | 44.16 | | 66.23 | | |  |  |
|  | aMT6s | Activity | | 77 | | single | | 160.75 | | 81.02 | | 179.97 | 594.60 | 0.00 | 594.61 | | 69.14 | | -13.85 | | 231.79 | | 1163.35 | | 240.44 | | 0.30 | | 0.01 | | 11.69 | | 23.38 | | 36.36 | | 63.64 | | |  |  |
|  | aMT6s | Skin temperature + activity | | 77 | | single | | 167.74 | | 92.20 | | 191.39 | 618.00 | 0.57 | 618.57 | | 65.50 | | -16.08 | | 246.55 | | 1193.29 | | 253.55 | | 0.27 | | 0.02 | | 10.39 | | 18.18 | | 36.36 | | 68.83 | | |  |  |
|  | aMT6s | Skin temperature | | 77 | | single | | 182.15 | | 66.42 | | 227.34 | 717.58 | 0.32 | 717.90 | | 86.88 | | 44.80 | | 278.66 | | 1406.89 | | 290.16 | | 0.36 | | 0.00 | | 15.58 | | 23.38 | | 46.75 | | 64.94 | | |  |  |

**Table S4.** Prediction error in minutes for network trained on all datasets, summarised by sleep-wake condition

|  | **Fixed Sleep** | **Habitual Sleep** | **Shift Work Diurnal** | **Shift Work Night Shift** |
| --- | --- | --- | --- | --- |
| n | 15 | 14 | 29 | 19 |
| Mean error | -11.27 | -30.39 | -12.77 | 291.73 |
| SD error | 77.40 | 76.19 | 103.24 | 302.48 |
| Mean absolute error | 62.43 | 60.13 | 67.71 | 353.99 |
| SD absolute error | 43.65 | 53.91 | 77.96 | 221.48 |
